# Identification of a Kupffer cell subset capable of reverting the T cell dysfunction induced by hepatocellular priming

**DOI:** 10.1016/j.immuni.2021.05.005

**Published:** 2021-09-14

**Authors:** Giorgia De Simone, Francesco Andreata, Camille Bleriot, Valeria Fumagalli, Chiara Laura, José M. Garcia-Manteiga, Pietro Di Lucia, Stefano Gilotto, Xenia Ficht, Federico F. De Ponti, Elisa B. Bono, Leonardo Giustini, Gioia Ambrosi, Marta Mainetti, Paola Zordan, Alexandre P. Bénéchet, Micol Ravà, Svetoslav Chakarov, Federica Moalli, Marc Bajenoff, Luca G. Guidotti, Florent Ginhoux, Matteo Iannacone

**Affiliations:** 1 Division of Immunology, Transplantation and Infectious Diseases, IRCCS San Raffaele Scientific Institute, 20132 Milan, Italy; 2 Vita-Salute San Raffaele University, 20132 Milan, Italy; 3 Singapore Immunology Network (SIgN), Agency for Science, Technology & Research (A^∗^STAR), 8A Biomedical Grove, Immunos Building #3-4, Biopolis, Singapore 138648; 4 Center for Omics Sciences, IRCCS San Raffaele Scientific Institute, 20132 Milan, Italy; 5 Aix Marseille University, CNRS, INSERM, CIML, Marseille 13288, France; 6 Shanghai Institute of Immunology, Shanghai JiaoTong University School of Medicine, 280 South Chongqing Road, Shanghai 200025, China; 7 Translational Immunology Institute, SingHealth Duke-NUS Academic Medical Centre, 169856, Singapore; 8 Experimental Imaging Centre, IRCCS San Raffaele Scientific Institute, 20132 Milan, Italy

**Keywords:** hepatitis B virus, CD8^+^ T cells, T cell dysfunction, liver, tolerance, Kupffer cells, interleukin-2, imaging, single cell, scRNA-seq

## Abstract

Kupffer cells (KCs) are highly abundant, intravascular, liver-resident macrophages known for their scavenger and phagocytic functions. KCs can also present antigens to CD8^+^ T cells and promote either tolerance or effector differentiation, but the mechanisms underlying these discrepant outcomes are poorly understood. Here, we used a mouse model of hepatitis B virus (HBV) infection, in which HBV-specific naive CD8^+^ T cells recognizing hepatocellular antigens are driven into a state of immune dysfunction, to identify a subset of KCs (referred to as KC2) that cross-presents hepatocellular antigens upon interleukin-2 (IL-2) administration, thus improving the antiviral function of T cells. Removing MHC-I from all KCs, including KC2, or selectively depleting KC2 impaired the capacity of IL-2 to revert the T cell dysfunction induced by intrahepatic priming. In summary, by sensing IL-2 and cross-presenting hepatocellular antigens, KC2 overcome the tolerogenic potential of the hepatic microenvironment, suggesting new strategies for boosting hepatic T cell immunity.

## Highlights

• KCs are required for *in vivo* reinvigoration of intrahepatically primed T cells by IL-2
• KCs respond to IL-2 and cross-present hepatocellular Ags
• Single-cell RNA-seq identifies two distinct populations of KCs
• KC2s have enriched IL-2 sensing machinery and Ag presentation capacity

## Graphical abstract

**Figure:**
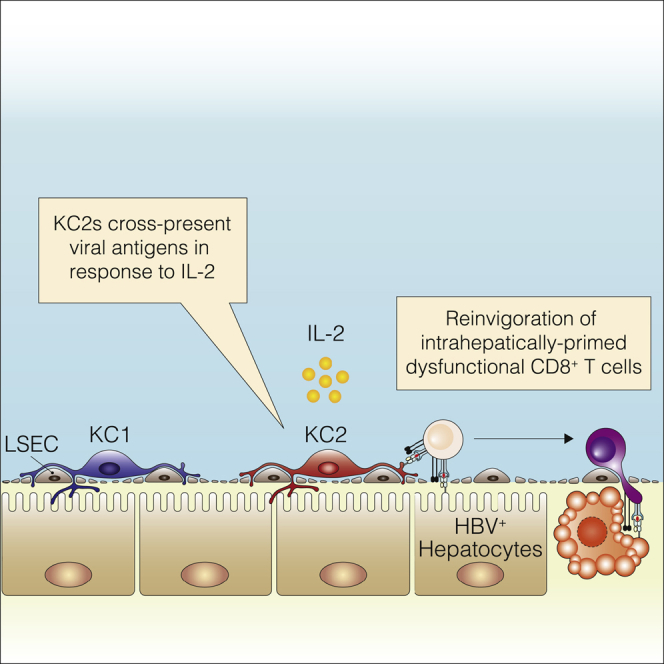

## Teaser

De Simone et al. delineate the mechanisms by which hepatocellularly primed HBV-specific CD8^+^ T cells acquire antiviral effector functions following IL-2 administration. These mechanisms rely on KCs and, in particular, on a hitherto unidentified KC subset, referred to as KC2, that is poised to respond to IL-2 and cross-present viral antigens.

## Introduction

The liver is peculiarly biased toward inducing immune tolerance, as exemplified by the acceptance of liver allografts across complete major histocompatibility complex (MHC) mismatch barriers or the propensity of hepatitis B virus (HBV) and other hepatotropic viruses such hepatitis C virus (HCV) to establish life-long persistent infections (Ficht and Iannacone, 2020; Wong et al., 2015). Liver tolerance involves a complex array of coordinated events that ultimately hinder the effector functions of intrahepatic lymphocytes (Ficht and Iannacone, 2020; Horst et al., 2016; Jenne and Kubes, 2013). For example, the unique anatomy and hemodynamics of the fenestrated and basement membrane-less liver capillaries (i.e., sinusoids), through which about one-third of all blood cells transit slowly every minute (Vollmar and Menger, 2009), allow circulating, intravascular T cells to sense MHC-antigen (Ag) complexes displayed by the non-professional Ag-presenting hepatocytes (Guidotti et al., 2015; Warren et al., 2006). Using mouse models of HBV infection, it has been recently shown that hepatocellular priming of virus-specific naive CD8^+^ T cells induces local activation and initial vigorous proliferation but eventually leads to the development of dysfunctional cells devoid of cytotoxic and antiviral activity (Bénéchet et al., 2019; Isogawa et al., 2013). The transcriptional signature of these cells does not obviously overlap with that of other known dysfunctional CD8^+^ T cell states such as exhaustion, and accordingly, CD8^+^ T cells primed by hepatocytes are not readily responsive to *in vivo* anti-PD-L1 treatment (Bénéchet et al., 2019). The notion that *in vivo* interleukin-2 (IL-2) administration overcomes this dysfunction (Bénéchet et al., 2019) not only illustrates that efficient hepatocellular priming can occur under specific conditions but also provides the opportunity to identify which cellular and molecular determinants drive immunogenic responses within the tolerogenic liver microenvironment.

Several formulations of IL-2 have been variably used in the past >25 years as a therapy to augment T cell responses against viral or tumor Ags (Blattman et al., 2003; Pol et al., 2020; West et al., 2013), and at the moment of writing, more than 40 clinical trials are evaluating the immune stimulatory potential of this cytokine in different oncological indications (https://www.clinicaltrials.gov). As the IL-2 functional pleiotropy has often driven undesired toxicity in the clinical setting (Pol et al., 2020), deconvoluting the biology responsible for its efficacy may help improve the therapeutic potential of IL-2-based strategies.

The IL-2 receptor consists of a heterocomplex of up to three subunits: α (CD25), β (CD122), and the common γ chain (CD132) (Pol et al., 2020). Although each receptor subunit can independently bind IL-2 with low affinity (*K*_d_ ~ 10^−8^ to 10^−7^ M), only the intermediate-affinity βγ dimeric (*K*_d_ ~ 10^−9^ M) and the high-affinity αβγ trimeric (*K*_d_ ~ 10^−11^ M) receptors mediate intracellular signal transduction (Pol et al., 2020). In addition to T cells and natural killer (NK) cells, myeloid cells have been reported to express the intermediate-affinity βγ receptor, with some dendritic cell (DC) subtypes displaying the three subunits of the IL-2 receptor (Bosco et al., 2000; Herr et al., 2014). However, the significance of IL-2 receptor expression by myeloid cells *in vivo* is controversial (Fukao and Koyasu, 2000; Kronin et al., 1998; Liang et al., 2012; Liao et al., 2013; Popov et al., 2008; Raeber et al., 2020). For instance, DCs may supply the α chain in *trans* (Wuest et al., 2011), thus supporting high-affinity binding of IL-2 to naive T cells undergoing priming. Other studies have suggested that DC production of CD25 quenches IL-2 in the outer T cell area of lymph nodes, thus guiding T cell differentiation (Li et al., 2016). Whether the IL-2-mediated reversal of the T cell dysfunction induced by hepatocellular priming is due to an exclusive effect of this cytokine on T cells or whether myeloid cells are involved is currently unknown.

## Results

### KCs are required for optimal *in vivo* reinvigoration of intrahepatically primed T cells by IL-2

**Figure 1:**
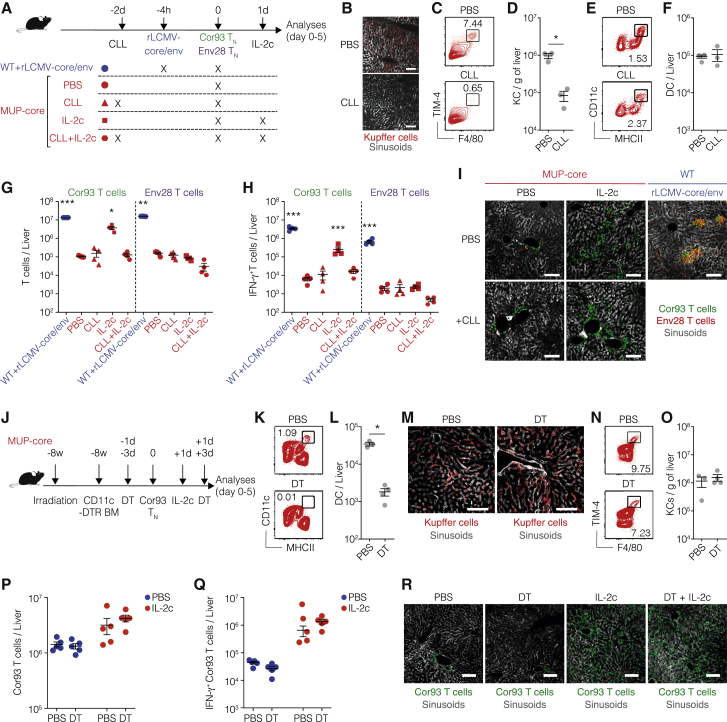
KCs are required for optimal *in vivo* reinvigoration of intrahepatically primed T cells by IL-2 (A) Schematic representation of the experimental setup. Cor93 and Env28 T_N_ (5 × 10^6^) were transferred into C57BL/6 × BALB/c F1 (WT) or MUP-core × BALB/c F1 (MUP-core) recipients. When indicated, mice were injected with 2.5 × 10^5^ infectious units of non-replicating rLCMV-core/env 4 h prior to T_N_ transfer. Selected MUP-core mice received clodronate liposomes (CLLs) and/or IL-2/anti-IL-2 complexes (IL-2c) at the indicated time points. Livers were collected and analyzed 5 days after T_N_ transfer. (B) Representative confocal immunofluorescence micrographs of liver sections from the indicated mice 48 h after CLL treatment. KCs were identified as F4/80^+^ cells and are depicted in red. Sinusoids were identified as Lyve-1^+^ cells and are depicted in gray. Scale bars represent 100 μm. (C and D) Representative flow cytometry plot (C) and absolute numbers (D) of KCs from the indicated mice 48 h after CLL treatment. KCs were identified as live, CD45^+^, TIM4^+^, F4/80^+^ cells. n = 3; ^∗^p < 0.05, one-tailed Mann-Whitney U test. (E and F) Representative flow cytometry plot (E) and absolute numbers (F) of dendritic cells (DCs; identified as live, MHC-II^hi^, CD11c^+^ cells) from the indicated mice 48 h after CLL treatment. n = 3. (G and H) Total numbers (G) and numbers of IFN-γ-producing (H) Cor93 and Env28 T cells in the livers of indicated mice. n = 4; ^∗^p < 0.05, ^∗∗^p < 0.01, ^∗∗∗^p < 0.001, one-way Brown-Forsythe and Welch ANOVA test with Dunnett correction for multiple comparisons. Each group was compared with control. Normal distribution was verified using the Shapiro-Wilk test. (I) Representative confocal immunofluorescence micrographs of liver sections from the indicated mice 5 days after T_N_ transfer. Cor93 T cells were identified as GFP^+^ cells and are depicted in green. Env28 T cells were identified as DsRed^+^ cells and are depicted in red. Sinusoids were identified as Lyve-1^+^ cells and are depicted in gray. Scale bars represent 100 μm. (J) Schematic representation of the experimental setup. MUP-core mice were lethally irradiated and reconstituted with CD11c^DTR^ bone marrow (BM). Eight weeks after BM reconstitution, 1 × 10^6^ Cor93 T_N_ were transferred. Indicated mice were treated with diphtheria toxin (DT) every 48 h starting from 3 days before T cell injection. Indicated mice received IL-2c 1 day after Cor93 T cell transfer. Livers were collected and analyzed 5 days after T_N_ transfer. (K and L) Representative flow cytometry plot (K) and absolute numbers (L) of DCs (identified as live, MHC-II^hi^, CD11c^+^ cells) from the indicated mice at the time of Cor93 T cell transfer (PBS, n = 3; DT, n = 4). ^∗^p < 0.05, one-tailed Mann-Whitney U test. (M) Representative confocal immunofluorescence micrographs of liver sections from the indicated mice 48 h after DT treatment. KCs were identified as F4/80^+^ cells and are depicted in red. Sinusoids were identified as Lyve-1^+^ cells and are depicted in gray. Scale bars represent 50 μm. (N and O) Representative flow cytometry plot (N) and absolute numbers (O) of KCs (identified as live, CD45^+^, TIM4^+^, F4/80^+^ cells) from the indicated mice at the time of Cor93 T cell transfer (PBS, n = 3; DT, n = 4). (P and Q) Total numbers (P) and numbers of IFN-γ-producing (Q) Cor93 T cells in the livers of the indicated mice. n = 5. (R) Representative confocal immunofluorescence micrographs of liver sections from the indicated mice 5 days after T_N_ transfer. Cor93 T cells were identified as CD45.1^+^ cells and are depicted in green. Sinusoids were identified as Lyve-1^+^ cells and are depicted in gray. Scale bars represent 100 μm. Data are representative of at least three independent experiments. See also Figure S1.

To shed light on the immune mechanisms underpinning the IL-2-mediated reinvigoration of intrahepatically primed T cells, we initially took advantage of transgenic mice that express a non-secretable version of the particulate HBV core protein under the transcriptional control of the hepatocyte-specific mouse major urinary protein (MUP) promoter (hereafter MUP-core mice) (Guidotti et al., 1994). These animals, like the HBV replication-competent transgenic mice described below, never develop spontaneous liver pathology, as the hepatocellular expression of the viral gene products occurs non-cytopathically, and endogenous T cells specific for these products are profoundly tolerant (Guidotti et al., 1994). As controls for proper CD8^+^ T cell differentiation into effector cells, we used wild-type (WT) mice transduced with recombinant, replication-defective lymphocytic choriomeningitis (LCMV)-based vectors (Flatz et al., 2010) targeting the HBV core and envelope proteins (rLCMV-core/env) to intrahepatic professional Ag-presenting cells (APCs) (i.e., Kupffer cells [KCs] and hepatic DCs) that are not natural targets of HBV (Bénéchet et al., 2019). Both groups of mice were injected with naive CD8^+^ TCR transgenic T cells (T_N_) specific for epitopes contained within the core and envelope proteins of HBV (Cor93 and Env28 T_N_, respectively) (Figure 1A) (Isogawa et al., 2013). One day after T_N_ injection, selected MUP-core mice received IL-2 immune complexes (IL-2c) consisting of IL-2 coupled with non-neutralizing IL-2-specific monoclonal antibodies (S4B6) that enhance the half-life of IL-2 *in vivo* (Boyman et al., 2006) (Figure 1A). To test whether IL-2c treatment had exclusively a direct effect on T_N_ or whether it required the presence of additional cells, we performed depletion experiments. We initially focused on KCs, as these cells are capable of inducing full effector differentiation of CD8^+^ T cells upon *in vivo* rLCMV transduction (Bénéchet et al., 2019). KCs were depleted through clodronate liposome (CLL) injection 2 days prior to T cell injection (Figure 1A). This treatment effectively depletes KCs while sparing hepatic DCs (Figures 1B–1F) (Bénéchet et al., 2019; Sitia et al., 2011). Consistent with previously published results (Bénéchet et al., 2019), Cor93 and Env28 T_N_ transferred to WT mice injected with rLCMV-core/env differentiated into bona fide effector cells that formed tight clusters scattered throughout the liver lobules; in contrast, Cor93 T cells transferred to MUP-core mice generated dysfunctional cells devoid of IFN-γ-producing ability that coalesced around portal tracts (Figures 1G–1I). IL-2c administration improved the capacity of Ag-specific Cor93 T cells to expand, differentiate into IFN-γ-producing cells and accumulate in clusters scattered throughout the liver lobules, but it had no effect on irrelevant Env28 T_N_ (Figures 1G–1I). Optimal *in vivo* reinvigoration of intrahepatically primed Cor93 T cells required the presence of KCs, as IL-2c treatment failed to improve T cell expansion, effector differentiation, and intraparenchymal cluster formation in CLL-treated mice (Figures 1G–1I). Similar results were obtained when recombinant IL-2 was used in place of IL-2c and when HBV replication-competent transgenic mice, which express all viral proteins in hepatocytes and secrete enveloped virions containing the HBV particulate core protein into the bloodstream, were used in place of MUP-core recipients (data not shown).

To confirm that hepatic DCs are not necessary for the optimal *in vivo* response to IL-2, we depleted this cell population by diphtheria toxin (DT) injection in MUP-core mice reconstituted with CD11c^DTR^ bone marrow (Figure 1J). This treatment significantly decreased the number of hepatic DCs while sparing KCs (Figures 1K–1O). DC depletion did not affect the capacity of IL-2 to promote expansion, effector differentiation, and intraparenchymal cluster accumulation of intrahepatically primed Cor93 T cells (Figures 1P–1R). Similarly, other phagocytic cells such as neutrophils and monocytes were found not to be involved in the response to IL-2, as neutrophil depletion (via anti-Ly6G Abs) or combined neutrophil and monocyte depletion (via anti-Gr1 Abs) did not affect the *in vivo* reinvigoration of intrahepatically primed T cells by IL-2 (Figure S1). Taken together, these results indicate that KCs are required for optimal *in vivo* reinvigoration of intrahepatically primed T cells by IL-2.

### KCs respond to IL-2 and cross-present hepatocellular Ags

**Figure 2:**
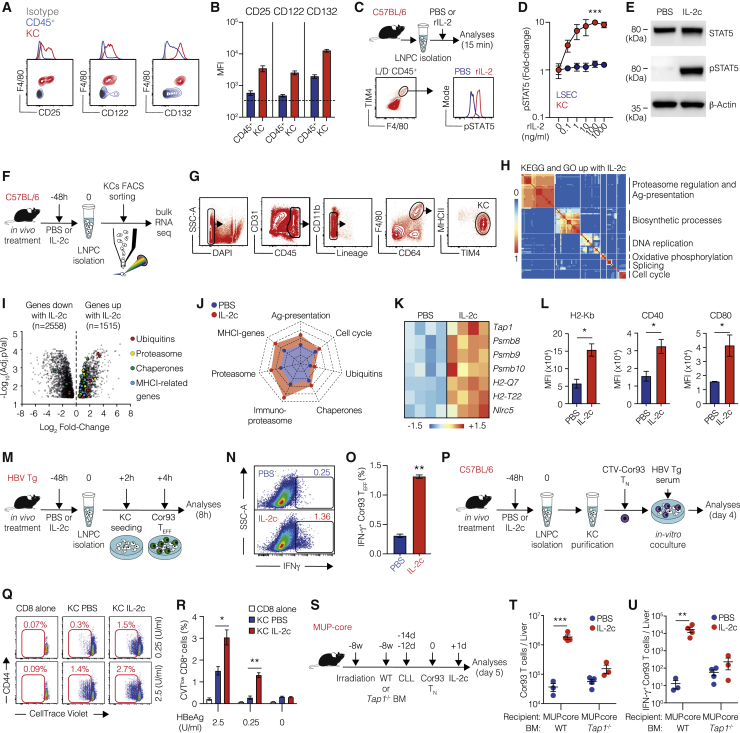
KCs respond to IL-2 and cross-present hepatocellular Ags (A) Representative flow cytometry plots of CD25 (left panel), CD122 (middle panel), and CD132 (right panel) expression on CD45^+^ (blue) and F4/80^+^ (red) cell populations in the livers of C57BL/6 mice. Isotype control is depicted in gray. (B) Mean fluorescent intensity (MFI) of CD25 (left), CD122 (middle), and CD132 (right) expression on live CD45^+^ (blue) and KCs (red; identified as live, CD45^+^, TIM4^+^, F4/80^+^ cells) cells in the livers of C57BL/6 mice. n = 3. (C) Schematic representation of the experimental setup. Liver non-parenchymal cells (LNPCs) were isolated from C57BL/6 mice and incubated *in vitro* for 15 min with increasing doses of rIL-2. pSTAT5 signal was analyzed on CD45^+^ F4/80^+^ TIM4^+^ cells (KCs) or CD31^+^ CD45^−^ cells (LSECs) using flow cytometry (representative plot of KCs at the bottom). (D) Fold change of STAT5 phosphorylation upon treatment with the indicated concentrations of rIL-2 in KCs (red dots) or LSECs (blue dots). n = 3; ^∗∗∗^p < 0.001, two-way ANOVA with Geisser-Greenhouse correction. Significance indicates time × column factor. (E) Immunoblot analysis of STAT5 and pSTAT5 in adherent KCs isolated from C57BL/6 mice and incubated *in vitro* with IL-2c or PBS. (F) Schematic representation of the experimental setup. C57BL/6 mice were treated *in vivo* with PBS or IL-2c. Forty-eight hours after treatment, liver non-parenchymal cells (LNPCs) were isolated, and RNA-seq was performed on flow cytometry-sorted KCs. (G) KC sorting strategy. KCs were identified as live, CD45^+^, Lineage^−^ (CD3, CD19, Ly6G, CD49b), F4/80^+^, CD64^+^, MHCII^int^, TIM4^+^ cells (n = 4 per group). (H) Clustering of top significant (EnrichR combined score > 100, false discovery rate [FDR] < 0.05) Gene Ontology biological processes and Kyoto Encyclopedia of Genes and Genomes (KEGG) pathways of processes upregulated in KCs upon *in vivo* IL-2c treatment. The thermal scale represents the Jaccard similarity coefficient between every gene set pair (blue representing a similarity coefficient of 0 and red a similarity coefficient of 1). (I) Volcano plot of RNA-seq results. The x axis represents the log_2_ fold change of differentially expressed genes (DEGs) upon IL-2c treatment, the y axis the −log_10_(FDR). Only DEGs with FDRs < 0.05 were considered. Genes belonging to specific biological process are highlighted in different colors (see also Figures S3A–S3E). (J) Radar plot of different biological processes. Each dimension of the radar plot is represented as the mean of the transcripts per kilobase million (TPM) of selected genes (see also Figures S3A–S3E), in PBS-treated (blue) and IL-2c-treated (red) samples. Values range from 0 to 350 TPM. (K) Heatmap of selected genes linked to Ag presentation that were upregulated in KCs upon IL-2c treatment. Values are *Z* scores, calculated from scaling by row the log_2_(TPM) values. (L) MFI of H2-K^b^, CD40, and CD80 expression on KCs (defined as live, CD45^+^, TIM4^+^, F4/80^+^ cells) 48 h after PBS or IL-2c treatment *in vivo*. n = 3; ^∗^p < 0.05, one-tailed Mann-Whitney U test. (M) Schematic representation of the experimental setup. HBV replication-competent transgenic mice (HBV Tg) were treated *in vivo* with PBS or IL-2c. After 48 h, liver non-parenchymal cells (LNPCs) were isolated, and KCs were seeded for 2 h and co-cultured with *in vitro*-differentiated Cor93 effector T cells (Cor93 T_E_). After 4 h, T cells were harvested and analyzed using flow cytometry. (N and O) Representative flow cytometry plot (N) and percentage (O) of IFN-γ producing Cor93 T_EFF_ cells in the indicated conditions. n = 3; ^∗∗^p < 0.01, one-tailed Mann-Whitney U test. (P) Schematic representation of the experimental setup. C57BL/6 mice were treated *in vivo* with PBS or IL-2c. After 48 h, LNPCs were isolated, and KCs were purified by immunomagnetic separation. Purified KCs were co-cultured with CellTrace violet (CTV)-labeled Cor93 T_N_. Serum from HBV replication-competent transgenic mice (containing the indicated concentrations of HBeAg) was added to the wells (note that HBeAg contains the Cor93 determinant). After 4 days, Cor93 T cells were harvested and analyzed using flow cytometry. (Q and R) Representative flow cytometry plots (Q) and percentages (R) of proliferating Cor93 T cells at the indicated conditions. ^∗^p < 0.05 and ^∗∗^p < 0.01, one-way Brown-Forsythe and Welch ANOVA test with Dunnett correction for multiple comparisons. Each group was compared with every other group within the same Ag dose. n = 3. Normal distribution was verified using the Shapiro-Wilk test. (S) Schematic representation of the experimental setup. MUP-core mice were lethally irradiated and reconstituted with WT or *Tap1*^−/−^ bone marrow (BM). Eight weeks after BM reconstitution, mice received two injection of clodronate liposomes (CLLs) to remove residual radio-resistant KCs. Two weeks after the last dose of CLL, 5 × 10^6^ Cor93 T_N_ were transferred. Indicated mice received IL-2c 1 day after Cor93 T cell transfer. Livers were collected and analyzed 5 days after Cor93 T_N_ transfer. (T and U) Total numbers (T) and numbers of IFN-γ-producing (U) Cor93 T cells in the livers of the indicated mice (MUP-core WT-PBS, n = 3; MUP-core WT-IL-2c, n = 4; MUP-core *Tap1*^−/−^-PBS, n = 4; MUP-core *Tap1*^−/−^-IL-2c, n = 4). ^∗∗^p < 0.01 and ^∗∗∗^p < 0.001, two-way ANOVA with Sidak’s multiple-comparison test. Data are representative of at least three independent experiments. See Figures S2 and S3 and Table S1.

Flow cytometric analyses revealed that a fraction of KCs expresses all three subunits of the IL-2 receptor (CD25, CD122, and CD132) (Figures 2A and 2B). We therefore investigated the effect of IL-2 treatment on KCs. To this end, we isolated liver non-parenchymal cells (LNPCs), including KCs, from C57BL/6 mice and stimulated them *ex vivo* with recombinant IL-2 (Figure 2C). We observed a dose-dependent increase in STAT5 phosphorylation in KCs but not in liver sinusoidal endothelial cells (LSECs) (Figure 2D). Similar results were obtained when IL-2c was used in place of IL-2, and STAT5 phosphorylation in KCs was confirmed by immunoblot analysis (Figure 2E). Of note, the IL-2-dependent fold change in STAT5 phosphorylation observed in KCs was ~10% than that observed in CD4^+^FoxP3^+^ splenic T regulatory cells (data not shown). Nevertheless, these data indicate that KCs express a functional IL-2 receptor capable of responding to IL-2 *in vitro*. To assess the consequences of IL-2 treatment on KCs *in vivo*, we treated C57BL/6 mice with IL-2c and then performed RNA sequencing (RNA-seq) analysis on flow cytometry-sorted KCs 48 h later (Figures 2F and 2G). A total of 4,073 differentially expressed genes (DEGs), 1,515 up- and 2,558 downregulated, were identified as significantly regulated by IL-2c (Table S1). Functional enrichment analysis of upregulated genes showed an increased transcription of genes involved mainly in Ag presentation and proteasomal processing, ribosomal RNA processing and splicing, DNA replication and cell cycle, and mitochondrial oxidative metabolism (Figure 2H; Figure S2; Table S1). Among the upregulated gene clusters, we focused on the Ag presentation pathway, which includes several macromolecular complexes composed of ubiquitins, chaperones, MHC-I, and proteasome subunits (Figures 2I–2K; Figures S3A–S3E) (Blum et al., 2013). Genes encoding for these protein families—specifically MHC-I-related proteins, immunoproteasome subunits, the transcription regulator of MHC-I genes *Nlrc5* (Kobayashi and van den Elsen, 2012) and the transporter associated with Ag processing 1 (*Tap1*)—were induced in KCs upon IL-2c treatment (Figures 2I–2K; Figures S3B–S3F). The upregulation of MHC-I and co-stimulatory molecules in KCs isolated from mice treated with IL-2c was confirmed at the protein level (Figure 2L). On the basis of these results, we reasoned that *in vivo* treatment with IL-2c might increase the cross-presentation ability of KCs. To test this possibility, we measured the capacity of *in vitro* differentiated Cor93-specific effector CD8^+^ T cells (Cor93 T_EFF_) to produce IFN-γ (as an indirect measure of Ag recognition) upon incubation with KCs isolated from control and IL-2c-treated HBV replication-competent transgenic mice (Figure 2M). Consistently with previously published data, baseline KC cross-presentation of the core protein in this experimental system at steady state was negligible (Figures 2N and 2O), despite KCs being constantly exposed to abundant HBV virions in the circulation. Cor93 T_N_ remained dysfunctional even when isolated from the liver of HBV replication-competent transgenic mice previously transferred with highly pathogenic Env28-specific effector CD8^+^ T cells (data not shown). This indicates that KC cross-presentation remains insignificant during acute liver inflammation, even though the inflammatory conditions potentially favor not only the uptake of HBV virions but also the phagocytosis of damaged hepatocytes containing the particulate HBV core protein. In spite of this, treating HBV replication-competent transgenic mice with IL-2c slightly but significantly increased the cross-presentation capacity of KCs incubated *in vitro* with Cor93 T_EFF_ cells (Figures 2N and 2O). We also assessed the ability of KCs isolated from IL-2-treated C57BL/6 mice to cross-prime HBV-specific naive CD8^+^ T cells exposed to the serum of HBV replication-competent transgenic mice *in vitro* (Figure 2P). Compared with KCs isolated from PBS-treated mice, KCs exposed to IL-2 *in vivo* induced a higher proliferation of Cor93 T_N_ in *in vitro* culture (Figures 2Q and 2R). Finally, to evaluate the *in vivo* relevance of our findings, we took advantage of MUP-core mice, which express only a non-secretable, particulate form of the HBV core protein and in which KC cross-presentation should depend on the uptake of the few hepatocytes that are known to be injured by Cor93 T_N_ transfer (Bénéchet et al., 2019). We generated MUP-core mice whose hematopoietic cells (including KCs) lack *Tap1* and therefore cannot express MHC-I and present Ags to CD8^+^ T cells. This was achieved by injection of either WT or *Tap1*^−/−^ bone marrow into irradiated MUP-core mice, followed by CLL treatment to deplete the residual radio-resistant KCs and allow the complete reconstitution of the entire KC compartment with bone marrow-derived cells (Sitia et al., 2011) (Figure 2S). Cor93 T_N_ injected into MUP-core mice whose hematopoietic cells (including KCs) lacked MHC-I had a much lower response to IL-2c than did Cor93 T_N_ injected into mice carrying Ag presentation-competent KCs (Figures 2T and 2U), suggesting that Cor93 T cells interacted with IL-2-stimulated KCs that cross-presented core protein-derived epitopes after the uptake of damaged hepatocytes. Taken together, these results indicate that optimal reinvigoration of intrahepatically primed CD8^+^ T cells by IL-2 requires the capacity of KCs to cross-present HBV Ags, possibly derived from circulating virions and/or damaged hepatocytes.

### Single-cell RNA-seq identifies two distinct populations of KCs among liver-resident macrophages

**Figure 3:**
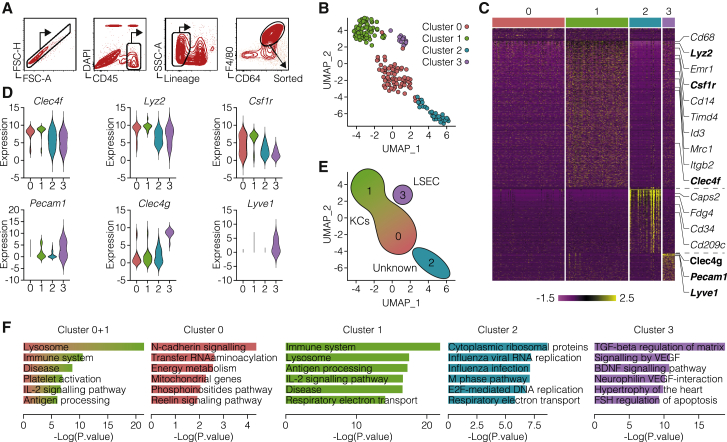
Single-cell RNA-seq identifies two distinct populations of KCs among liver-resident macrophages (A) Sorting strategy for liver macrophages. Liver macrophages are defined as live, CD45^+^, Lineage^−^ (CD3, CD19, Ly6G, CD49b), CD64^+^, F4/80^+^ cells. (B) UMAP projection of sorted cells. Each dot corresponds to a single cell, colored according to the unbiased clusters identified: cluster 0 (red, 68 cells), cluster 1 (green, 59 cells), cluster 2 (blue, 30 cells), and cluster 3 (purple, 12 cells). (C) Heatmap of normalized and scaled expression values of the 2,811 marker genes identifying the four clusters. Genes highlighted on the right are representative of each cluster. Color coding of the bar on the top of the heatmap as in (B). (D) Violin plots showing the normalized expression profile of selected genes differentially expressed in the four clusters. (E) Cell type annotation of the four clusters on the basis of the identified markers. (F) Pathway analysis of each cluster. Enriched pathways (Huang et al., 2019) are ordered by p value, and the most biologically informative among the top ten are shown. See also Figure S4, Table S2, and Table S3.

Next, we asked whether the IL-2-responsive KCs represent a distinct subpopulation. To this end, we used high-dimensional single-cell RNA-seq (scRNA-seq) to characterize KC heterogeneity at steady state. We flow cytometry-sorted live CD45^+^ Lineage^−^ CD64^+^ F4/80^+^ liver macrophages from C57BL/6 mice (Figure 3A), isolated RNA, and generated transcriptional profiles for each cell (n = 169) using the Smart-seq2 pipeline (Picelli et al., 2014). This dataset was analyzed using Seurat (Stuart et al., 2019), and four main cell clusters were identified and visualized using uniform manifold approximation and projection (UMAP) (Becht et al., 2018) (Figure 3B). Cluster 0 (n = 68) and cluster 1 (n = 59) cells showed higher expression of classical KC-associated gene markers, such as *Clec4f*, *Lyz2*, and *Csf1r* (Figures 3C and 3D; Table S2). Pathway analysis of their respective gene markers yielded immunological pathways and processes typical of macrophages and professional APCs and were thus considered bona fide KCs (Figures 3E and 3F; Table S3). Cells in cluster 2 (n = 30) expressed genes such as *Cd34*, *Cd209c*, and *Fgd4* but low amounts of macrophage genes (Figures 3C and 3D), while among their specific markers we found a large number of ribosomal and non-coding genes. They also showed a smaller number of transcripts detected per cell and a higher percentage of mitochondrial genes, indicating a high fraction of apoptotic cells in this population, and hence were excluded from subsequent analyses (Figure 3E and data not shown). Cells in cluster 3 (n = 12) expressed genes associated with endothelial cells, including *Pecam1* (CD31), *Clec4g*, *Lyve1* (Figures 3C and 3D), and *Kdr* (VGFR2) (Table S2); in addition, their specific markers were enriched in endothelial cell processes (Figure 3F; Table S3), arguing for contamination of sorted cells with LSECs (Figure 3E). Although both cluster 0 and cluster 1 showed expression of KC markers, they were clearly distinguished by the expression of many genes (Figure 3C). Of note, compared with cells in cluster 0, we found that cells in cluster 1 were enriched in genes associated with Ag processing, cross-presentation, and IL-2 signaling pathway (Figure 3F; Tables S2 and S3). Among the DEGs, we initially used *Mrc1* (CD206) and *Lamp2* (CD107b) (Figures S4A and S4B; Table S2) as a first approach to identify and flow cytometry-sort the two KC populations. An *ad interim* bulk RNA-seq analysis of the two populations revealed *Esam* (ESAM) as highly differentially expressed (Figure S4C).

### A KC subset with enriched IL-2 sensing machinery and Ag presentation capacity can be identified

**Figure 4:**
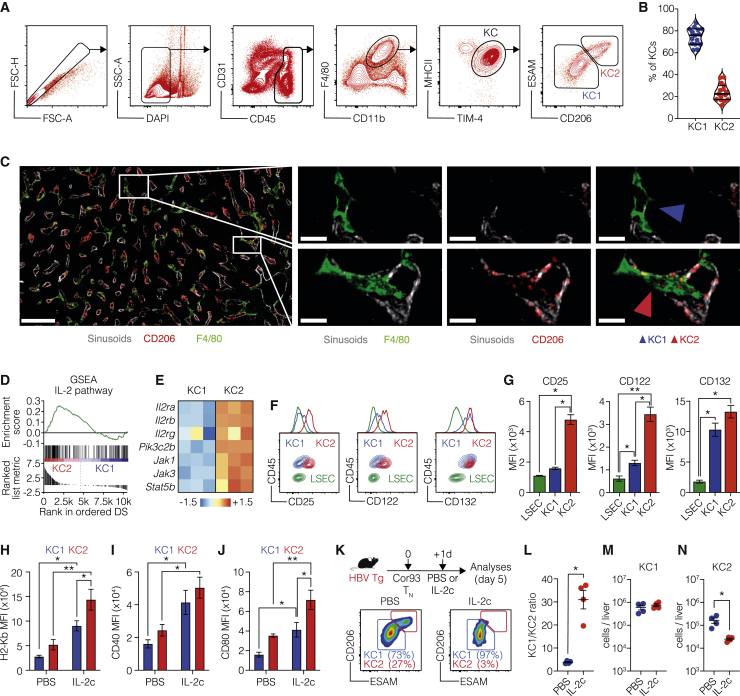
Identification of a KC subset with enriched IL-2 sensing machinery (A) Representative flow cytometry plot of KC1 and KC2 gating strategy. KCs are identified as live, CD45^+^, CD11b^int^, F4/80^+^, MHCII^+^, TIM4^+^ liver non-parenchymal cells. KC1 are defined as ESAM^−^ CD206^−^ KCs. KC2 are defined as ESAM^+^ CD206^+^ KCs. (B) Relative representation of KC1 and KC2 percentages in the liver of C57BL/6 mice (n = 15). (C) Representative confocal immunofluorescence micrographs of liver sections from C57BL/6 mice. Sinusoids were identified as CD38^+^ cells and are depicted in white. CD206^+^ cells are depicted in red and F4/80^+^ cells in green. Scale bars represent 50 or 10 μm (see also Video S1). (D) GSEA relative to the IL-2 pathway enrichment in KC2 (red) and KC1 (blue) samples. Genes were pre-ranked on the basis of the log_2_ fold change between KC2 and KC1. (E) Heatmap representing the relative expression of the IL-2 receptor signaling components in KC1 and KC2 isolated from C57BL/6 mice (n = 3 per group). Values in log_2_(TPM) were scaled by row across samples (*Z* score). (F and G) Representative flow cytometry plots (F) and MFI (G) of CD25, CD122, and CD132 expression in KC1, KC2, and LSEC (defined as live, CD45^−^, CD31^+^ cells) in C57BL/6 mice (n = 3 per group). ^∗^p < 0.05 and ^∗∗^p < 0.01, two-way ANOVA with Sidak’s multiple-comparison test. (H–J) MFI of H2-Kb (H), CD40 (I), and CD80 (J) expression on KC1 (blue) and KC2 (red) 48 h after PBS or IL-2c treatment *in vivo* (n = 3 per group). ^∗^p < 0.05 and ^∗∗^p < 0.01, two-way ANOVA with Sidak’s multiple-comparison test. Test is performed comparing PBS versus IL-2c treatment and KC1 versus KC2. (K) Schematic representation of the experimental setup. HBV Tg mice were injected with 1 × 10^6^ Cor93 T_N_ cells. Mice were treated with PBS or IL-2c 1 day after Cor93 T_N_ transfer. Livers were collected and analyzed 5 days after T_N_ transfer. Representative flow cytometry plots (bottom) of KC1 and KC2 in the livers upon PBS (left) or IL-2c (right) treatment. (L–N) Ratio between KC1 and KC2 (L) and absolute numbers of KC1 (M) and KC2 (N) in the liver of PBS-treated (blue) or IL-2c-treated (red) mice. n = 4; ^∗^p < 0.05, one-tailed Mann-Whitney U test. Data are representative of at least three independent experiments. See also Figure S5, Table S4, and Video S1.

On the basis of these data, we designed a panel of markers for use in conventional flow cytometry to identify these KC subpopulations and validate the aforementioned high-throughput approach. The CD45^+^ F4/80^+^ CD11b^int^ TIM-4^+^ KC population split into CD206^−^ESAM^−^ (hereafter KC1; ~70%–85% of total KCs) and CD206^+^ESAM^+^ (hereafter KC2; ~15%–30% of total KCs) cells (Figures 4A and 4B). Imaging analyses confirmed the presence of two distinct KC subpopulations (Figure 4C; Video S1). Importantly, RNA-seq analyses on bulk KC1 and KC2 sorted from C57BL/6 mice confirmed that KC2 are enriched in IL-2 signaling components (IL-2 receptor subunits and molecules implicated in intracellular signal transduction) (Figures 4D and 4E; Table S4). Higher expression of the IL-2 receptor subunits, MHC-I, and co-stimulatory molecules in KC2 was confirmed at the protein level using flow cytometry analysis (Figures 4F–4J). Together, the data suggest that KC2 are better equipped than KC1 to respond to IL-2 and increase their capacity to cross-present hepatocellular Ags. Thus, one might predict that IL-2 treatment might render KC2 more sensitive than KC1 to CD8^+^ T cell-mediated killing. To test this hypothesis, we treated HBV replication-competent transgenic mice with IL-2c 24 h after Cor93 T_N_ cell injection and checked the KC1/KC2 ratio 4 days later (Figure 4K). Consistent with the hypothesis that IL-2 preferentially increased the capacity of KC2 to cross-present hepatocellular Ags and thus rendered them more sensitive to CD8^+^ T cell-mediated killing, we found that KC2 almost completely disappeared in Cor93 T cell-injected HBV transgenic mice treated with IL-2c (Figures 4L–4N). Notably, neither IL-2c treatment alone (in the absence of Cor93 T_N_ cell transfer) nor severe liver inflammation (induced by Cor93 T_EFF_) altered the KC1/KC2 ratio (Figure S5).

Video S1. Confocal immunofluorescence histology of KC1 and KC2, related to Figure 4 F4/80^+^ cells are depicted in green, CD206^+^ cells in red and CD38^+^ cells in white. Examples of KC1 (F4/80^+^ CD206^-^ cells), KC2 (F4/80^+^CD206^+^ cells) and LSECs (CD38^+^ CD206^+^ cells) are shown.6

### KC2 are required for the optimal restoration of intrahepatically primed, dysfunctional CD8^+^ T cells by IL-2

**Figure 5:**
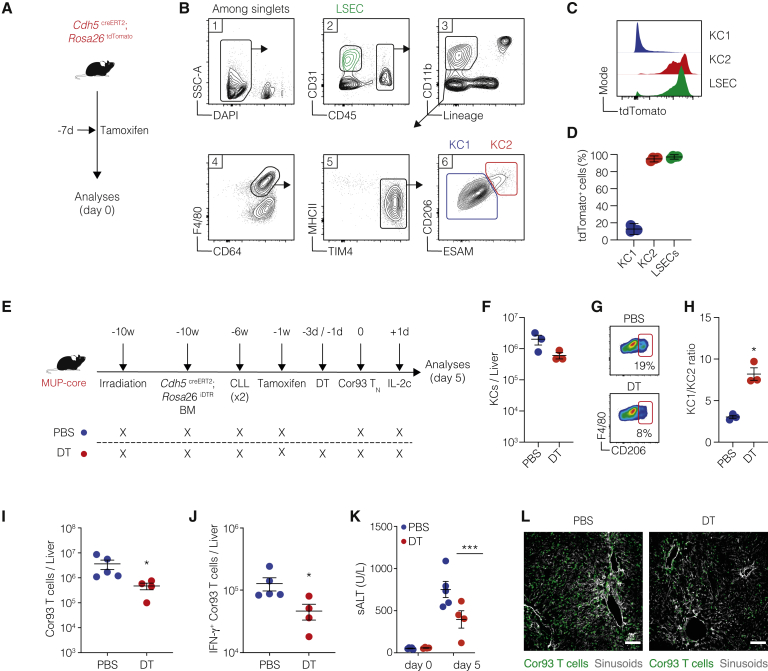
KC2 are required for the optimal restoration of intrahepatically primed, dysfunctional CD8^+^ T cells by IL-2 (A) Schematic representation of the experimental setup. Cdh5 ^CreERT2^; Rosa26 ^tdTomato^ mice were treated with tamoxifen, and livers were collected and analyzed 7 days after treatment. (B) Gating strategy for KC1, KC2, and LSECs. (C and D) Representative histograms (C) and percentage (D) of tdTomato expression on of KC1 (blue) and KC2 (red) and LSECs (green) (n = 3). (E) Schematic representation of the experimental setup. MUP-core mice were lethally irradiated and reconstituted with *Cdh5*^*creERT2*^*; Rosa26*^*iDTR*^ bone marrow (BM). Four weeks later, mice received two injections of clodronate liposomes (CLLs) to remove residual radio-resistant KCs. Nine weeks after BM reconstitution, mice were treated once with 5 mg of tamoxifen by oral gavage. Mice were treated with diphtheria toxin (DT) every 48 h starting 3 days before Cor93 T_N_ injection (1 × 10^6^ cells/mouse). Indicated mice received IL-2c 1 day after Cor93 T_N_ transfer. Livers were collected and analyzed 5 days after Cor93 T_N_ transfer. (F) Absolute numbers of total KCs (defined as live, CD45^+^, TIM4^+^, F4/80^+^ cells) in the liver of PBS (blue) or DT (red) treated mice. (G) Representative flow cytometry plots of KC1 (CD206^−^ KCs) and KC2 (CD206^+^ KCs) populations gated on total KCs (live, CD45^+^, TIM4^+^, F4/80^+^ cells) in the liver of the indicated mice at the time of T_N_ injection. (H) Ratio between KC1 and KC2 in the liver of PBS-treated (blue) or DT-treated (red) mice. n = 3; ^∗^p < 0.05, one-tailed Mann-Whitney U test. (I and J) Total numbers (I) and numbers (J) of IFN-γ-producing Cor93 T cells in the livers of the indicated mice. PBS, n = 5; DT, n = 4. ^∗^p < 0.05, two-tailed Mann-Whitney U test. (K) Amount of ALT in the serum of the indicated mice at the indicated time points. PBS, n = 5; DT, n = 4. ^∗∗∗^p < 0.001, two-way ANOVA with Sidak’s multiple-comparison test. (L) Representative confocal immunofluorescence micrographs of liver sections from the indicated mice 5 days after Cor93 T_N_ transfer. Cor93 T cells were identified as CD45.1^+^ cells and are depicted in green. Sinusoids were identified as CD38^+^ cells and are depicted in gray. Scale bars represent 100 μm. Data are representative of two independent experiments.

We next sought to generate a model in which KC2 could be selectively depleted to assess their role in the cross-presentation of hepatocellular Ags upon *in vivo* IL-2 treatment. We took advantage of the observation that KC2 (but not KC1) express the endothelial cell marker VE-cadherin (encoded by *Cdh5*) (Figures 5A–5D) to establish a system allowing inducible depletion of KC2 but not endothelial cells. This was achieved by (1) injecting *Cdh5*^*cre*ERT2^; Rosa26^iDTR^ bone marrow into irradiated MUP-core mice, (2) depleting the residual radio-resistant KCs by CLL to allow the complete reconstitution of the entire KC compartment with bone marrow-derived cells, (3) inducing DTR expression in KC2 by tamoxifen administration, and finally (4) depleting KC2 by DT injection prior to Cor93 T_N_ transfer followed by IL-2c treatment (Figure 5E). DT treatment caused a ~75% decrease in KC2 (Figures 5G and 5H) and resulted in a lower ability of Cor93 T cells to proliferate and differentiate into cytotoxic effector cells clustered throughout the liver lobule in response to IL-2c (Figures 5I–5L). These data indicate that KC2 are required for the optimal reinvigoration of intrahepatically primed T cells by IL-2.

## Discussion

Here, we have delineated the mechanisms by which hepatocellularly primed HBV-specific CD8^+^ T cells acquire antiviral and pathogenic effector functions following the exogenous administration of IL-2. These mechanisms rely on KCs and, in particular, on a hitherto unidentified subset of KCs, referred to as KC2, that is poised to respond to IL-2 and cross-present viral Ags contained within circulating virions or within hepatocytes.

The observation that DCs, generally regarded as the main cross-presenting APCs *in vivo* (Joffre et al., 2012), are dispensable for the optimal reinvigoration of intrahepatically primed T cells by IL-2 remains to be explored but is consistent with the lack of a functional IL-2 receptor expression by DCs (Raeber et al., 2020).

The results reported here are noteworthy considering that steady-state KC cross-presentation of HBV Ags is an inefficient process that cannot be increased by liver inflammation, hepatocellular death, or the administration of therapeutic monoclonal antibodies directed against HBsAg leading to the generation of circulating immune complexes (Fumagalli et al., 2020). Our data do not rule out a direct effect of IL-2 on T cells; however, they indicate that optimal *in vivo* reinvigoration of intrahepatically primed T cells by IL-2 depends on the presence of KC2. The extent to which the reported increase in cross-presentation of hepatocellular Ags by KCs in response to *in vivo* IL-2 administration is the result of direct IL-2 sensing by KCs or an indirect effect remains to be determined.

The location of CD8^+^ T cell priming during natural HBV infection is unclear. Immunological dogma holds that naive CD8^+^ T cells initially encounter cognate Ag in secondary lymphoid organs where cross-priming by professional APCs of circulating virions and subviral particles promotes the differentiation into effector cells endowed with liver homing potential. Yet there is no direct experimental evidence indicating that secondary lymphoid organs are the priming site for CD8^+^ T cells in HBV-infected humans or chimpanzees, and the idea behind their involvement has been supported by the assumption that the intrahepatic priming of naive CD8^+^ T cells (especially in the presence of high Ag load) should promote functional impairment of these cells. On the basis of these considerations, it is generally believed that priming of HBV-specific naive CD8^+^ T cells in the liver cannot promote viral clearance but, rather, contribute to the establishment of a persistent infection. However, on the basis of the results presented here, it is tempting to speculate that during acute HBV infection, the liver is fully competent to sustain the priming of HBV-specific effector CD8^+^ T cells endowed with antiviral activity, provided it occurs in the presence of high local concentrations of IL-2 that increase KC2 cross-presentation. A putative source of IL-2 in this scenario might be intrahepatic Ag-specific effector CD4^+^ T cells. In support of this hypothesis, which cannot be tested in humans because of the limitations of collecting liver biopsies in acutely infected patients, are the observations that (1) CD4^+^ T cell depletion in chimpanzees prior to infection precludes effective T cell priming and causes persistent infection with minimal immunopathology (Asabe et al., 2009), and (2) detection of CD8^+^ T cells in the liver of HBV-infected chimpanzees coincides with the hepatic detection of CD4^+^ T cells (Guidotti et al., 1999).

In parallel to the present study, we have used high-dimensional single-cell sequencing, mass cytometry, and flow cytometry, coupled with *in vivo* fate-mapping models to perform an in-depth characterization of KC2 at steady state (Bleriot et al., 2021, this issue of *Immunity*). These analyses have revealed a specific metabolic role for KC2 in regulating glucose homeostasis and oxidative stress (Bleriot et al., 2021). Future studies should be directed at identifying the signals required for KC2 development and maintenance and explore the potential role of these cells in other diseases affecting the liver.

We envision that strategies aimed at targeting IL-2 to KC2 should be considered for the treatment of chronic HBV infection and for other conditions similarly requiring to overcome the tolerogenic potential of the hepatic microenvironment. Such strategies might include liposome or nanoparticle-based formulations targeting KC2-expressed surface Ags as well as integrase-defective third-generation lentiviral vectors exploiting combinations of transcriptional and post-transcriptional microRNA-mediated control (Bénéchet et al., 2019).

### Limitations of study

The therapeutic implications of our study rely on the existence of an IL-2-responsive, cross-presenting KC population in humans. Of note, recent publications suggest the existence of a human KC2-like subset that expresses at least some of the human orthologs of the KC2-specific genes identified here (Aizarani et al., 2019; MacParland et al., 2018; Ramachandran et al., 2019; Wu et al., 2020). Future studies are certainly warranted to dissect human KC heterogeneity and function in the healthy as well as in the diseased liver.

## STAR★Methods

### Key resources table

**Table:** 

REAGENT or RESOURCE	SOURCE	IDENTIFIER
**Antibodies**		

PE-CF594 anti-mouse CD3e	BD Biosciences	BD Biosciences Cat# 562286; RRID: AB_11153307
eFluor 450 anti-mouse CD4	eBioscience	eBioscienceCat# 48-0042-82; RRID: AB_468865
PB rat anti-mouse CD8a	BD Biosciences	BD Biosciences Cat# 558106; RRID: AB_397029
BV650 anti-mouse/human CD11b	BioLegend	BioLegend Cat# 101239; RRID: AB_11125575
PE-CF594 rat anti-mouse CD19	BD Biosciences	BD Biosciences Cat# 562291; RRID: AB_11154223
PE/Cyanine7 anti-mouse CD25	BioLegend	BioLegend Cat# 102015; RRID: AB_312864
BV605 anti-mouse CD31	BioLegend	BioLegend Cat# 102427; RRID: AB_2563982
BUV395 anti-mouse CD45	BD Biosciences	BD Biosciences Cat# 564279; RRID: AB_2651134
BV711 anti-mouse CD64	BioLegend	BioLegend Cat# 139311; RRID: AB_2563846
APC/Cyanine7 anti-mouse F4/80	BioLegend	BioLegend Cat# 123117; RRID: AB_893489
AF700 anti-mouse I-A/I-E	BioLegend	BioLegend Cat# 107621; RRID: AB_493726
PE/cyanine7 anti-mouse Tim-4	BioLegend	BioLegend Cat# 130010; RRID: AB_2565719
FITC TIM-4	Biorbyt	BiorbytCat# orb103599
AF647 anti-mouse CD69	BioLegend	BioLegend Cat# 104517; RRID: AB_492848
APC/cyanine7 anti-mouse CD45.1	BioLegend	BioLegend Cat# 110715; RRID: AB_313504
AF647 anti-Mouse IFN-γ	BD Biosciences	BD Biosciences Cat# 557735; RRID: AB_396843
PE anti-mouse CD11c	BioLegend	BioLegend Cat# 117308; RRID: AB_313777
PE/cyanine7 anti-mouse I-Ab	BioLegend	BioLegend Cat# 116420; RRID: AB_10575296
AF647 anti-Stat5 (pY694)	BD Biosciences	BD Biosciences Cat# 612599; RRID: AB_399882
PE-cyanine7 anti-mouse/rat foxp3	Thermo Fisher Scientific	Thermo Fisher Scientific Cat# 25-5773-82; RRID: AB_891552
PE anti-mouse CD122	BioLegend	BioLegend Cat# 123210; RRID: AB_940617
PE anti-mouse CD132	BioLegend	BioLegend Cat# 132306; RRID: AB_2280163
APC rat anti-mouse CD40	BD Biosciences	BD Biosciences Cat# 558695; RRID: AB_1645224
PE hamster anti-mouse CD80	BD Biosciences	BD Biosciences Cat# 553769; RRID: AB_395039
BV650 mouse anti-mouse H-2Kb	BD Biosciences	BD Biosciences Cat# 742861; RRID: AB_2741103
PE anti-mouse ESAM	BioLegend	BioLegend Cat# 136203; RRID: AB_1953300
AF647 anti-mouse CD206	BioLegend	BioLegend Cat# 141712; RRID: AB_10900420
PE-CF594 rat anti-mouse Ly-6G	BD Biosciences	BD Biosciences Cat# 562700; RRID: AB_2737730
PE anti-mouse Ly-6C antibody	BioLegend	BioLegend Cat# 128008; RRID: AB_1186132)
PE-CF594 rat anti-mouse CD49b	BD Biosciences	BD Biosciences Cat# 562453; RRID: AB_11153857
PE anti-mouse CD107b	Thermo Fisher Scientific	Thermo Fisher Scientific Cat# 12-5989-82; RRID: AB_466103
AF488 anti-mouse F4/80	BioLegend	BioLegend Cat# 123120; RRID: AB_893479
APC anti-mouse CD206	BioLegend	BioLegend Cat# 141708; RRID: AB_10900231
AF594 anti-mouse CD38	BioLegend	BioLegend Cat# 102725; RRID: AB_2566435
AF647 anti-mouse CD45.1	BioLegend	BioLegend Cat# 110720; RRID: AB_313491
Purified anti-mouse CD38	BioLegend	BioLegend Cat# 102702; RRID: AB_312923
F4/80 monoclonal antibody	Thermo Fisher Scientific	Thermo Fisher Scientific Cat# MF48000; RRID: AB_10376289
InVivoMAb anti-mouse CD16/CD32	Bio X Cell	Bio X Cell Cat# BE0307; RRID:AB_2736987
LYVE-1 antibody	Novus Biological	Novus Biological Cat# NB600-1008; RRID:AB_10000497
Alexa fluor 488, chicken anti-rabbit IgG (H+L) cross-adsorbed secondary antibody	Thermo Fisher Scientific	Thermo Fisher Scientific Cat# A-21441; RRID: AB_2535859
Alexa fluor 568, goat anti-rat IgG (H+L) cross-adsorbed secondary antibody	Thermo Fisher Scientific	Thermo Fisher Scientific Cat# A-11077; RRID: AB_2534121
Alexa fluor 488, chicken antirRat IgG (H+L) cross-adsorbed secondary asntibody	Thermo Fisher Scientific	Thermo Fisher Scientific Cat# A-21470; RRID: AB_2535873
Rabbit mAb anti-stat5	Cell Signaling Technology	Cell Signaling Technology Cat# 94205; RRID: AB_2737403
XP Rabbit mAb anti-phospho-Stat5 (Tyr694)	Cell Signaling Technology	Cell Signaling Technology Cat# 4322; RRID: AB_10544692
Peroxidase AffiniPure Goat Anti-Rabbit IgG (H+L)	Jackson ImmunoResearch Labs	Jackson ImmunoResearch Labs Cat# 111-035-003; RRID: AB_2313567
InVivoMAb anti-mouse IL-2	Bio X Cell	Bio X Cell Cat# BE0043-1; RRID: AB_1107705
InVivoMab anti-mouse Ly6G	Bio X Cell	Bio X Cell Cat# BE0075-1; RRID: AB_1107721
InVivoMab anti-mouse Ly6G/Ly6C	Bio X Cell	Bio X Cell Cat# BE0075; RRID: AB_10312146

**Recombinant viral vectors**		

rLCMV-core/env	(Bénéchet et al., 2019)	N/A

**Chemicals, peptides, and recombinant proteins**		

*Recombinant mouse IL-2 protein*	R and D Systems	R and D Systems Cat# 402-ML-500/CF
Clodronate liposomes	Liposoma	Liposoma Cat# C-025
Diphtheria toxin	Millipore	Millipore Cat# 322326
Tamoxifen	Sigma	Sigma Cat# T5648-5G
Corn oil	Sigma	Sigma Cat# C8267-500ML
Viobility 405/520 fixable dye	Miltenyi Biotec	Miltenyi Cat# 130-109-816
LIVE/DEAD fixable far-red dye	Thermo Fisher Scientific	Thermo Fisher Scientific, Cat# L34973
DAPI	Thermo Fisher Scientific	Thermo Fisher Scientific Cat# D1306; RRID:AB_2629482
Phosflow perm buffer III	BD Biosciences	BD Biosciences Cat# 558050; RRID: AB_2869118
O.C.T.	Bio Optica	Bio Optica Cat# 05-9801
FluorSave reagent	Millipore	Millipore Cat# 345789

**Critical commercial assays**		

Foxp3 / transcription factor staining buffer set	Thermo Fisher Scientific	Thermo Fisher Scientific, Cat# 00-5523-00
CellTrace violet cell proliferation kit	Thermo Fisher Scientific	Thermo Fisher Scientific, Cat# C34571
Anti-F4/80 MicroBeads UltraPure, mouse	Miltenyi Biotec	Miltenyi Biotec Cat# 130-110-443, RRID: AB_2858241
EasySep mouse naive CD8+ T cell isolation kit	Stem Cell technologies	Stem Cell technologiesCat# 19858
Nextera XT DNA library preparation kit	Illumina	Illumina Cat# FC-131-1024
DNA high sensitivity reagent kit	Perkin Elmer Labchip	Perkin Elmer Labchip Cat# CLS760672
Arcturus picoPure RNA isolation kit	Thermo Fisher Scientific	Thermo Fisher Scientific Cat# KIT0204
ERCC RNA spike-In mix	Thermo Fisher Scientific	Thermo Fisher Scientific Cat# 4456740
ReliaPrep RNA miniprep system	Promega	Promega Cat# PRZ6012
TURBO DNA-free kit	Thermo Fisher Scientific	Thermo Fisher Scientific Cat# AM1907
Qubit RNA HS assay kit	Thermo Fisher Scientific	Thermo Fisher Scientific Cat# Q32852
NovaSeq reagent kits	Illumina	Illumina Cat# 20028312
Clarity Western ECL substrate kit	Bio-Rad	Bio-Rad Cat# 1705060S

**Deposited data**		

Bulk RNaseq data	This manuscript	NCBI Gene Expression Omnibus (GEO) accession GEO: GSE152211
Single-cell RNaseq data	This manuscript	NCBI Gene Expression Omnibus (GEO) accession GEO: GSE168989

**Experimental models: organisms/strains**		

Mouse: C57BL/6	Charles River	C57BL/6 colony
Mouse: BALB/c	Charles River	BALB/c colony
Mouse: CBy.PL(B6)-Thya/ScrJ	The Jackson Laboratory	Stock No: 005443
Mouse: C57BL/6-Tg(CAG-EGFP)1Osb/J	The Jackson Laboratory	Stock No: 003291
Mouse: B6.Cg-Gt(ROSA)26Sortm14(CAG-tdTomato)Hze/J	The Jackson Laboratory	Stock No: 007914
Mouse: B6.Cg-Tg(CAG-DsRed^∗^MST)1Nagy/J	The Jackson Laboratory	Stock No: 006051
Mouse: B6.129S2-Tap1tm1Arp/J	The Jackson Laboratory	Stock No: 002458
Mouse: B6.FVB-1700016L2RikTg(Itgax-DTR/EGFP)57Lan/J	The Jackson Laboratory	Stock No: 004509
Mouse: C57BL/6-Gt(ROSA)26Sortm1(HBEGF)Awai/J	The Jackson Laboratory	Stock No: 007900
Mouse: Tg(Cdh5-cre/ERT2)1Rha	The Jackson Laboratory	MGI:3848982
Mouse: MUP-core 50 [MC50]	(Guidotti et al., 1994)	Internal colony
Mouse: lineage 1.3.32	(Guidotti et al., 1995)	Internal colony
Mouse: lineage BC10.3	(Isogawa et al., 2013)	Internal colony
Mouse: lineage 6C2.36	(Isogawa et al., 2013)	Internal colony

**Software and algorithms**		

FlowJo V10	FlowJo	https://www.flowjo.com/
RSEM tool	(Li and Dewey, 2011)	https://deweylab.github.io/RSEM/
Seurat (v3.2.2)	(Stuart et al., 2019)	https://satijalab.org/seurat/
Enrichr	(Kuleshov et al., 2016)	https://maayanlab.cloud/Enrichr/
featureCounts	(Liao et al., 2019)	https://www.rdocumentation.org/packages/Rsubread/versions/1.22.2/topics/featureCounts
edgeR	(Robinson et al., 2010)	http://bioconductor.org/packages/release/bioc/html/edgeR.html
STAR aligner	(Dobin et al., 2013)	https://github.com/alexdobin/STAR
LIMMA R package	(Ritchie et al., 2015)	https://bioconductor.org/packages/release/bioc/html/limma.html
pheatmap R	Raivo Kolde	https://www.rdocumentation.org/packages/pheatmap/versions/1.0.12/topics/pheatmap
fmsb R	Minato Nakazawa	https://cran.r-project.org/web/packages/fmsb/index.html
Cytoscape	Cytoscape	https://cytoscape.org
GseaPreranked	(Subramanian et al., 2005)	https://gsea-msigdb.github.io/gseapreranked-gpmodule/v6/index.html
homologene R package	Ogan Mancarci, Leon French	https://cran.r-project.org/web/packages/homologene/index.html
Prism 9	GraphPad software	https://www.graphpad.com/scientific-software/prism
BD FACSDiva V8	BD Biosciences	https://www.bdbiosciences.com/us/instruments/research/software/flow-cytometry-acquisition/bd-facsdiva-software/m/111112/overview
CytExpert	Beckman Coulter	https://www.beckman.com/flow-cytometry/instruments/cytoflex/software
Leica Application Suite X (LAS X)	Leica Microsystem	https://www.leica-microsystems.com/products/microscope-software/p/leica-las-x-ls/
Imaris bitplane	Imaris	https://imaris.oxinst.com/products/imaris-for-cell-biologists?gclid=Cj0KCQiAgomBBhDXARIsAFNyUqOQMD64vZvZMyBoHWFOYRm_ZPxHWLb_tWDl0pGjii8ZVNDkW-UNtRgaAnhfEALw_wcB
Fiji-Imagej	Imagej	https://imagej.net/Fiji/Downloads

**Other**		

FACS CANTO II	BD Bioscience	N/A
CytoFLEX LX	Beckman Coulter	N/A
FACSAria Fusion	BD Bioscience	N/A
Illumina HiSeq 4000 system	Illumina	N/A
Agilent Bioanalyser	Agilent	N/A
UVItec	Eppendorf	N/A
SP5 or SP8 confocal microscope	Leica Microsystem	N/A
Vet abcTM	scil	N/A

### Resource availability

#### Lead contact

Further information and requests for resources and reagents should be directed to and will be fulfilled by the Lead Contact, Matteo Iannacone (iannacone.matteo@hsr.it).

#### Materials availability

This study did not generate new unique reagents.

### Experimental model and subject details

#### Mice

C57BL/6, CD45.1 (inbred C57BL/6), BALB/c, Thy1.1 (CBy.PL(B6)-*Thy*^*a*^/ScrJ), β-actin-GFP [C57BL/6-Tg(CAG-EGFP)1Osb/J], Ai14(RCL-tdT)-D [B6.Cg-Gt(ROSA)26Sortm14(CAG-tdTomato)Hze/J ], β-actin-DsRed [B6.Cg-Tg(CAG-DsRed^∗^MST)1Nagy/J], *Tap1*^−/−^ (B6.129S2-*Tap1*^*tm1Arp*^/J), CD11c^DTR^ [B6.FVB-*1700016L2Rik*^*Tg(Itgax-DTR/EGFP)57Lan*^/J], ROSA26^iDTR^ [C57BL/6-Gt(ROSA)26Sortm1(HBEGF)Awai/J], Cdh5^CreERT2^ [Tg(Cdh5-cre/ERT2)1Rha] mice were purchased from Charles River or The Jackson Laboratory. MUP-core transgenic mice (lineage MUP-core 50 [MC50], inbred C57BL/6, H-2^b^), that express the HBV core protein in 100% of the hepatocytes under the transcriptional control of the mouse major urinary protein (MUP) promoter, have been previously described (Guidotti et al., 1994). HBV replication-competent transgenic mice (lineage 1.3.32, inbred C57BL/6, H-2^b^), that express all of the HBV Ags and replicate HBV in the liver at high viral copies without any evidence of cytopathology, have been previously described (Guidotti et al., 1995). In indicated experiments, MUP-core and HBV replication-competent transgenic mice were used as C57BL/6 x BALB/c H-2^bxd^ F1 hybrids. Cor93 TCR transgenic mice (lineage BC10.3, inbred CD45.1), in which > 98% of the splenic CD8^+^ T cells recognize a K^b^-restricted epitope located between residues 93-100 in the HBV core protein (MGLKFRQL), have been previously described (Isogawa et al., 2013). Env28 TCR transgenic mice (lineage 6C2.36, inbred Thy1.1 BALB/c), in which ~83% of the splenic CD8^+^ T cells recognize a L^d^-restricted epitope located between residues 28–39 of HBsAg (IPQSLDSWWTSL), have been previously described (Isogawa et al., 2013). For imaging experiments Cor93 transgenic mice were bred against β-actin-GFP, while Env28 transgenic mice were bred against β-actin-DsRed mice (inbred BALB/c). Bone marrow (BM) chimeras were generated by irradiation of MUP-core or C57BL/6 mice with one dose of 900 rad and reconstitution with the indicated BM; mice were allowed to reconstitute for at least 8 weeks before experimental manipulations. Mice were housed under specific pathogen-free conditions and entered experiments at 8-10 weeks of age. In all experiments, mice were matched for age, sex and (for the 1.3.32 animals) serum HBeAg concentration before experimental manipulations. All experimental animal procedures were approved by the Institutional Animal Committee of the San Raffaele Scientific Institute and are compliant with all relevant ethical regulations.

#### Viruses and viral vectors

Replication-incompetent LCMV-based vectors encoding HBV core and envelope proteins (rLCMV-core/env) were generated, grown and titrated as previously described (Bénéchet et al., 2019). Mice were injected intravenously (i.v.) with 2.5 × 10^5^ infectious units of rLCMV vector 4h before CD8^+^ T cell injection. All infectious work was performed in designated BSL-2 or BSL-3 workspaces, in accordance with institutional guidelines.

### Method details

#### Naive T cell isolation, adoptive transfer and *in vivo* treatments

Mice were adoptively transferred with 5 × 10^6^ or 1 × 10^6^ HBV-specific naive CD8^+^ TCR transgenic T cells isolated from the spleens of Cor93 and/or Env28 TCR transgenic mice, as described (Bénéchet et al., 2019). IL-2/anti-IL-2 complexes (IL-2c) were prepared by incubating 1.5 μg of rIL-2 (R&D Systems) with 50 μg anti-IL-2 mAb (clone S4B6-1, BioXcell) per mouse, as previously described (Boyman et al., 2006). Mice were injected with IL-2c intraperitoneally (i.p.) one day after T cell transfer, unless otherwise indicated. In indicated experiments, naive CD8^+^ T cells from the spleens of Cor93 TCR transgenic mice were differentiated *in vitro* for 7-9 days into effector cells prior to adoptive transfer (1 × 10^7^ cells), or *in vitro* co-culture, as described (Bénéchet et al., 2019; Guidotti et al., 2015). In indicated experiments, Kupffer cells (KCs) were depleted by intravenous injection of 200 μL of clodronate-containing liposomes (Liposoma) 2 days prior to T cell injection, as described (Bénéchet et al., 2019), unless otherwise indicated. In indicated experiments, mice were injected i.p. with 200 μg of anti-Ly6G depleting antibody (clone 1A8, BioXcell) one day before and one day after T cell transfer. In indicated experiments, mice were injected intravenously (i.v.) with 200 μg of anti-Gr1 depleting antibody (clone RB6-8C5, BioXcell) every 48h starting from 3 days before T cell transfer. In indicated experiments, C57BL/6 or MUP-core mice were lethally irradiated and reconstituted for at least 8 weeks with BM from CD11c-DTR mice; dendritic cells were subsequently depleted by injecting i.p. 20 ng per gram of mouse of diphtheria toxin (Millipore) every 48h starting from 3 days before T cell transfer. In indicated experiments, MUP-core mice were lethally irradiated and reconstituted for at least 8 weeks with BM from C57BL/6 or *Tap1*^−/−^ mice. To achieve full reconstitution of Kupffer cells from donor-derived BM, mice were injected with 200 μL of clodronate-containing liposomes 28 and 31 days after BM injection. In indicated experiments, MUP-core mice were lethally irradiated and reconstituted for at least 8 weeks with BM from *Cdh5*^CreERT2^; Rosa26^iDTR^; Rosa26^tdTomato^; CX3CR1^GFP^ mice. To achieve full reconstitution of Kupffer cells from donor-derived BM, mice were injected with 200 μL of clodronate-containing liposomes 28 and 31 days after BM injection. To induce the expression of the Cre recombinase, mice were treated with 5 mg of Tamoxifen (Sigma) by oral gavage in 200 μL of corn oil one week before further manipulations. KC2 were depleted subsequently by injecting i.p. 20 ng per gram of mouse of diphtheria toxin (Millipore) 3 days and 1 day prior to T cell transfer.

#### Cell isolation and flow cytometry

Single-cell suspensions of liver, spleen and blood were generated as described (Bénéchet et al., 2019). Kupffer cell isolation was performed as described (Bénéchet et al., 2019). All flow cytometry stainings of surface-expressed and intracellular molecules were performed as described (De Giovanni et al., 2020). Cell viability was assessed by staining with Viobility 405/520 fixable dye (Miltenyi, #130-109-816), LIVE/DEAD Fixable Far-Red dye (Invitrogen, # L34973) or DAPI (Invitrogen, # D1306). Abs used included: anti-CD3 (clone: 145-2C11, Cat#562286, BD Biosciences), anti-CD4 (clone: RM4-5, Cat #48-0042-82, eBioscience), anti-CD8a (clone: 53-6.7, Cat# 558106, BD Biosciences), anti-CD11b (clone: M1/70, Cat#101239), anti-CD19 (clone: 1D3, Cat#562291 BD Biosciences), anti-CD25 (clone: PC61, Cat#102015), anti-CD31 (clone: 390, Cat#102427), anti-CD45 (clone: 30-F11, Cat#564279 BD Biosciences), anti-CD64 (clone: X54-5/7.1, Cat#139311), anti-F4/80 (clone: BM8, Cat#123117), anti-I-A/I-E (clone: M5/114.15.2, Cat#107622), anti-TIM4 (clone: RTM4-54 Cat#130010), anti-TIM4 (polyclonal, Cat#orb103599 Biorbyt), anti-CD69 (clone: H1.2F3, Cat# 104517), anti-CD45.1 (clone: A20, Cat#110716), anti-IFN-γ (clone: XMG1.2, Cat# 557735 BD Biosciences), anti-CD11c (clone: N418, Cat# 117308), anti-I-Ab (clone: AF6-120.1, Cat# 116420), anti-Stat5 pY694 (clone: 47, Cat# 612599 BD Biosciences), anti-Foxp3 (clone FJK-16 s, Cat#25-5773-82 Thermofisher), anti-CD122 (clone TM-B1 Cat#123210), anti-CD132 (clone TUgm2 Cat#132306), anti-CD40 (clone 3/23 Cat#558695 BD Biosciences), anti-CD80 (clone 1610A1 Cat#553769 BD Biosciences), anti-H2-K^b^ (clone AF6-88.5 Cat#742861 BD Biosciences), anti-ESAM (clone 1G8/ESAM, Cat#136203), anti-CD206 (clone C068C2, Cat#141712), anti-Ly6G (clone 1A8, Cat #562700 BD Biosciences), anti-Ly6C (clone HK1.4, Cat# 128008), anti-CD49b (clone DX5, Cat#562453 BD Biosciences), anti CD107b (clone M3/84, Cat #12-5989-82 eBioscience). All Abs were purchased from BioLegend, unless otherwise indicated. Recombinant dimeric H-2L^d^:Ig and H-2K^b^:Ig fusion proteins (BD Biosciences) complexed with peptides derived from HBsAg (Env28-39) or from HBcAg (Cor93-100), respectively, were prepared according to the manufacturer’s instructions. Dimer staining was performed as described (Iannacone et al., 2005). Flow cytometry staining for phosphorylated STAT5 was performed using Phosflow Perm Buffer III (BD Bioscience), following the manufacturer’s instructions. Flow cytometry staining for Foxp3 was performed using Foxp3/Transcription Factor Staining Buffer Set (eBioscience), following the manufacturer’s instructions. In indicated experiments, cells were stained with CellTrace™ Violet cell proliferation kit (CTV, Invitrogen), following manufacturer’s instructions. All flow cytometry analyses were performed in FACS buffer containing PBS with 2 mM EDTA and 2% FBS on a FACS CANTO II (BD Bioscience) or CytoFLEX LX (Beckman Coulter) and analyzed with FlowJo software (Treestar).

#### Cell purification

For the experiment described in Figure 2, KCs were sorted from liver non-parenchymal cells as live, lineage negative (CD3^-^, CD19^-^, Ly6G^-^, CD49b^-^), CD45^+^, CD11b^int^, F4/80^+^, CD64^+^, MHCII^+^, TIM4^+^ cells. For the experiment described in Figure 3, single cells were sorted from liver non-parenchymal cells as live, CD45^+^, lineage negative (CD3^-^, CD19^-^, Ly6G^-^, CD49b^-^), F4/80^+^, CD64^+^ cells. For the experiment described in Figure 4, KCs were sorted from liver non-parenchymal cells as live, CD45^+^, CD11b^int^, F4/80^+^, MHCII^+^, TIM4^+^ cells. Among total KCs, KC1 were sorted as CD206^-^ ESAM^-^ cells and KC2 as CD206^+^, ESAM^+^ cells. Total KCs, KC1 and KC2 were flow cytometry-sorted with a 100 μm nozzle at 4°C on a FACSAria Fusion (BD) cell sorter in a buffer containing PBS with 2% FBS. Cells were always at least 98% pure (data not shown). In indicated experiments, F4/80^+^ cells were purified from liver non-parenchymal cells by positive immunomagnetic separation (Miltenyi Biotec, #130-110-443), according to the manufacturer’s instructions. In indicated experiments, CD8^+^ T cells were purified from splenocytes using EasySep™ kit (StemCell # 19858), according to the manufacturer’s instructions.

#### Single-cell RNA-seq

Single cells were sorted on a 96-well plate and cDNA libraries were generated using the Smart-seq v2 protocol (Picelli et al., 2014) with the following modifications: i) 1mg/ml BSA Lysis buffer (Ambion® Thermo Fisher Scientific, Waltham, MA, USA); ii) use of 200 pg cDNA with 1/5 reaction of Illumina Nextera XT kit (Illumina, San Diego, CA, USA). The length distribution of the cDNA libraries was monitored using a DNA High Sensitivity Reagent Kit on the Perkin Elmer Labchip (Perkin Elmer, Waltham, MA, USA). All samples were subjected to an indexed paired-end sequencing run of 2x151 cycles on an Illumina HiSeq 4000 system (Illumina, San Diego, CA, USA) (298 samples/lane). The RSEM tool (Li and Dewey, 2011) was used to perform Transcript Per Million (TPM) normalization starting from FASTQ files.

Single cell data analysis was performed using Seurat (v3.2.2) (Stuart et al., 2019). 169 cells were obtained after applying a filter to the TMP matrix of at least 200 genes expressed per cell and only genes expressed in at least 3 cells were retained. TPM expression was further normalized and scaled using the SCTransform function, and Umap reduction was then applied on first 12 Principal Components after running PCA. Unbiased clustering was made using the FindClusters function in Seurat with default parameters and a resolution value of 1. Specific markers for the different unbiased clusters were found using the function FindAllmarkers or FindMarkers in Seurat with default parameters and were then used for functional enrichment analysis with the online tool *Enrichr* (Kuleshov et al., 2016).

The plots showing normalized expression values with a color scale on top of Umap plots (on Figure S4) and the Violin plots of specific genes were produced with FeaturePlot and VlnPlot Seurat functions, respectively.

#### RNA purification and RNA-seq library preparation

Bulk RNA-seq on CD206^-^CD107b^-^ and CD206^+^CD107b^+^ cells (shown in Figure S4): between 20,000 and 50,000 cells were flow cytometry-sorted using CD206 (*Mrc1*) and CD107b (*Lamp2*) to identify CD206^-^, CD107b^-^ and CD206^+^, CD107b^+^ cells. Total RNA was extracted using Arcturus PicoPure. RNA Isolation kit (Arcturus. Thermo Fisher Scientific, Waltham, MA, USA) according to manufacturer’s protocol. All Mouse RNAs were analyzed on Agilent Bioanalyser (Agilent, Santa Clara, CA, USA) for quality assessment with RNA Integrity Number (RIN) range from 5.8 to 6.7 and median of RIN 6.4. cDNA libraries were prepared using 2 ng of total RNA and 1ul of a 1:50,000 dilution of ERCC RNA Spike in Controls (Ambion. Thermo Fisher Scientific, Waltham, MA, USA) using the Smart-Seq v2 protocol (Picelli et al., 2014) with the following modifications: i) addition of 20 μM TSO; ii) use of 200 pg cDNA with 1/5 reaction of Illumina Nextera XT kit (Illumina, San Diego, CA, USA). The length distribution of the cDNA libraries was monitored using a DNA High Sensitivity Reagent Kit on the Perkin Elmer Labchip (Perkin Elmer, Waltham, MA, USA). All samples were subjected to an indexed paired-end sequencing run of 2x151 cycles on an Illumina HiSeq 4000 system (Illumina) (25 samples/lane)

Bulk RNA-seq experiment on total KCs (shown in Figure 2) and bulk RNA-seq experiment on sorted KC1 and KC2 (shown in Figure 4): flow cytometry-sorted KCs, KC1 and KC2 were lysed in ReliaPrep RNA Cell Miniprep System (Promega #Z6011) and total RNA was isolated following manual extraction. DNA digestion was performed with TURBO DNA-free Kit (Invitrogen #AM1907). RNA was quantified with Qubit RNA HS Assay Kit (Invitrogen # Q32852) and analysis of its integrity was assessed with Agilent RNA 6000 Pico Kit (Agilent #5067-1513) on a Bioanalyser instrument. 6 RNA samples of sorted KC1 and KC2, were processed with the “SMART-seq Ultra Low Input 48” library protocol in order to obtain 30.0M clusters of fragments of 1x100nt of length through NovaSeq 6000 SP Reagent Kit (100 cycles).

#### RNA-seq bioinformatics analysis

Bulk RNA-seq experiment on CD206^-^, CD107b^-^ and CD206^+^, CD107b^+^ cells (shown in Figure S4): raw reads were obtained and mapped to the mouse genome build GRCm38. Gene counts were generated using featureCounts (part of the R subread package) (Liao et al., 2019) with GENCODE version M9 annotations. Differential Expression Analysis genes (DEGs) and MA plots were performed using the R package *edgeR* (Robinson et al., 2010).

Bulk RNA-seq experiment on total KCs (shown in Figure 2) and on sorted KC1 and KC2 (shown in Figure 4): raw reads were aligned to mouse genome build GRCm38 using STAR aligner (Dobin et al., 2013). Read counts per gene were then calculated using featureCounts based on GENCODE gene annotation version M16. Read counts were subject to log2 TPM (transcript per million) normalization to account for transcript length and library size.

Only genes with a TPM value higher than 1 in at least 4 (for the total KC experiment in Figure 2) or 3 (for the KC2 versus KC1) samples were considered for following analysis. Differentially Expressed Genes (DEGs) between groups treated with IL-2c and PBS were identified by generating a linear model using LIMMA R package (Ritchie et al., 2015). Only DEGs with an adjusted P value < 0.05 (using Benjamini Hochberg correction method) were selected for further analysis. For the final KC2 versus KC1 comparison an additional |logFC| > 1 filter was applied.

#### Functional enrichment analysis

Bulk RNA-seq analysis of the experiment described in Figure 2: of the 4073 significant (FDR < 0.05) identified DEGs between control (PBS) and treated (IL-2c) samples, 1515 were upregulated and 2558 were downregulated. Those were subject to a functional enrichment analysis using the *EnrichR* R package (Kuleshov et al., 2016). Both the up- and the downregulated DEGs were checked for any biological signature enrichment in both the Gene Ontology Biological Process Database and the Kyoto Encyclopedia of Genes and Genomes for Mouse. After merging the results for the two databases, 858 significant (FDR < 0.05) Terms were identified, of which 428 were derived from the upregulated DEGs and 430 from the downregulated ones. In order to select the top enriched terms, only those with a high Combined Score (-log(p value) ^∗^ Odds Ratio) were considered. Based on the distribution of the Combined Score in the upregulated terms and in the downregulated ones, a threshold of 100 was chosen for the former, while a threshold of 30 for the latter.

#### Clustering of upregulated terms

For visualization and analysis, both upregulated and downregulated terms were subject to a clustering algorithm, in order to identify the most prominent biological signatures. Briefly, a Jaccard Index Similarity score was calculated for each pair set of terms, based on the DEGs annotated for each term, using an *in-house* developed script. Next, terms were clustered using a hierarchical clustering method, using as distance measure the Pearson correlation between the calculated Jaccard Index Similarity scores. An arbitrary number of clusters was selected and manually annotated based on the terms present. To visualize the result, the *pheatmap* R package was used.

#### Radar plots visualization

Radar plots were generated using the *fmsb* R package. Different sets of genes were selected based on literature analysis, defining different biological processes. For each category, the mean TPM expression for each gene within samples (separately for control and treated samples) was calculated. Next, the mean between all the genes belonging to a category was calculated and used as the value to represent the dimension in the radar plot.

#### Network plot visualization

Network plot (Figure S3F) was built using *Cytoscape* software (V 3.8.0 for MacOS). Briefly, starting from *EnrichR* tables (Table S1), a matrix defining every pair of term-gene was generated, and used as a node list input for *Cytoscape*.

#### Gene set enrichment analysis

Gene Set Enrichment Analysis (GSEA) from bulk RNA-seq of KC1 and KC2 (Figure 4) was performed using the GseaPreranked Java tool (Subramanian et al., 2005) using pre-ranked Log2 fold changes between KC2 and KC1 populations in expressed genes. HALLMARK_IL2_STAT5_SIGNALING Gene Set contained in MsigDB (Broad Institute) (Liberzon et al., 2015), Version 6. Since the gene set is based on human genes, mouse orthologs in humans were identified using the *homologene* R package (https://cran.r-project.org/web/packages/homologene/index.html).

#### Immunoblot analysis

Immunoblot on plated KCs was performed as described (Zordan et al., 2018). Primary Abs include anti-STAT5 and anti-pSTAT5 (Tyr694) (rabbit; Cell Signaling Technology #8215) and β-actin (polyclonal; Abcam ab228001). As secondary Ab horseradish peroxidase-conjugated goat anti-rabbit IgG (Jackson ImmunoResearch, Cat# 111-035-003) was used. Reactive proteins were visualized using a Clarity Western ECL substrate kit (Bio-Rad), and exposure was performed using UVItec (Cambridge MINI HD, Eppendorf). Images were acquired by NineAlliance software.

#### Confocal immunofluorescence histology and histochemistry

Confocal microscopy analysis of livers was performed as described (Guidotti et al., 2015). For confocal images of KC1 and KC2, C57BL/6 mice were injected i.v. with 2 μg of anti-F4/80 Alexa Fluor 488 (BioLegend, #123120) and 2 μg of anti-CD206 APC (BioLegend, #141708) 10 minutes before harvesting the liver. The liver was fixed overnight in PBS with 4% paraformaldehyde and subsequently incubated for 24h in PBS with 30% sucrose. Next, liver lobes were embedded in O.C.T (Killik Bio-Optica) and cut at −14°C into 60 μm thick sections with a cryostat. Sections were blocked for 15 min with blocking buffer (PBS, 0.5% BSA, 0.3 % Triton) and stained for 1h at room temperature (RT) with anti-CD38 Alexa Fluor 594 (BioLegend, #102725) in wash/stain buffer (PBS, 0.2% BSA, 0.1% triton). Sections were then washed twice for 5 min, stained with DAPI (Sigma) for 5 min, washed again and mounted for imaging with FluorSave Reagent (Millipore). For additional confocal imaging, the following primary Abs were used for staining: anti-CD45.1 AF647 (110720, BioLegend), anti-F4/80 (BM8, Invitrogen), anti-Lyve-1 (NB600-1008, Novus Biological), anti-CD38 (102702, BioLegend). The following secondary Abs were used for staining: Alexa Fluor 488-, Alexa Fluor 514-, Alexa Fluor 568-conjugated anti-rabbit or anti-rat IgG (Life Technologies). Image acquisition was performed with a 63x oil-immersion or 20x objective on an SP5 or SP8 confocal microscope (Leica Microsystem). To minimize fluorophore spectral spillover, the Leica sequential laser excitation and detection modality was used. Where necessary to compensate for uneven slide illumination, fluorescent intensity of layers was normalized using Imaris normalize Layers tool. Where necessary, autofluorescence was filtered from the image by channel subtraction of a deep red autofluorescent channel from APC signal with the Imaris Channel Arithmetics tool.

#### Biochemical analyses

The extent of hepatocellular injury was monitored by measuring serum alanine aminotransferase (sALT) activity at multiple time points after treatment, as previously described (Guidotti et al., 2015). Serum HBeAg was measured by enzyme-linked immunosorbent assays (ELISA), as previously described (Guidotti et al., 2015). Blood cell counts were measured by Vet abc (scil).

### Quantification and statistical analysis

Results are expressed as mean ± s.e.m. All statistical analyses were performed in Prism (GraphPad Software), and details are provided in the figure legends. Normality of data distribution was tested in all graphs with a Shapiro-Wilk or D’Agostino & Pearson normality test and parametric tests were chosen only when normality could be confirmed for each dataset. One-tailed test were chosen over two-tailed test when basic biology dictates that the change between the control and treatment group can only occur into one direction (e.g., in cell depletion experiments, where the number of cells will be decreased in the treatment versus the control group). Comparisons are not statistically significant unless indicated.

## Data Availability

Bulk RNA-seq data generated during this study have been deposited in the Gene Expression Omnibus (GEO) with the accession code GEO: GSE152211. Single Cell RNA-seq data generated during this study have been deposited in the Gene Expression Omnibus (GEO) with the accession code GEO: GSE168989.

## Supplemental information

Document S1. Figures S1–S5

Table S1. Differential gene expression and EnrichR tables of functional enrichment of DEGs up- and downregulated in Kupffer cells upon in vivo IL-2c treatment, related to Figure 2 Experimental set up is described in Figure 2F. Genes expressed (log2TPM > 1 in at least 4 samples) were processed using LIMMA (Ritchie et al., 2015).

Table S2. Markers of identified clusters in the scRNA-seq experiment, related to Figure 3

Table S3. EnrichR tables of functional enrichment of marker genes in the scRNA-seq experiment, related to Figure 3 (cells in clusters 0 and 1 analyzed together and separately)

Table S4. Differential gene expression in KC2 relative to KC1, related to Figure 4 Genes expressed (log2TPM > 1 in at least 3 samples) were processed using LIMMA (Ritchie et al., 2015).

Document S2. Article plus supplemental information

## References

[bib1] Aizarani N., Saviano A., Sagar, Mailly L., Durand S., Herman J.S., Pessaux P., Baumert T.F., Grün D. (2019). A human liver cell atlas reveals heterogeneity and epithelial progenitors. Nature.

[bib2] Asabe S., Wieland S.F., Chattopadhyay P.K., Roederer M., Engle R.E., Purcell R.H., Chisari F.V. (2009). The size of the viral inoculum contributes to the outcome of hepatitis B virus infection. J. Virol..

[bib3] Becht E., McInnes L., Healy J., Dutertre C.-A., Kwok I.W.H., Ng L.G., Ginhoux F., Newell E.W. (2018). Dimensionality reduction for visualizing single-cell data using UMAP. Nat. Biotechnol..

[bib4] Bénéchet A.P., De Simone G., Di Lucia P., Cilenti F., Barbiera G., Le Bert N., Fumagalli V., Lusito E., Moalli F., Bianchessi V. (2019). Dynamics and genomic landscape of CD8^+^ T cells undergoing hepatic priming. Nature.

[bib5] Blattman J.N., Grayson J.M., Wherry E.J., Kaech S.M., Smith K.A., Ahmed R. (2003). Therapeutic use of IL-2 to enhance antiviral T-cell responses in vivo. Nat. Med..

[bib6] Bleriot C., Barreby E., Dunsmore G., Ballaire R., Chakarov S., Ficht X., De Simone G., Andreata D., Fumagalli V., Guo W. (2021). A subset of Kupffer cells regulates metabolism through the expression of CD36. Immunity.

[bib7] Blum J.S., Wearsch P.A., Cresswell P. (2013). Pathways of antigen processing. Annu. Rev. Immunol..

[bib8] Bosco M.C., Curiel R.E., Zea A.H., Malabarba M.G., Ortaldo J.R., Espinoza-Delgado I. (2000). IL-2 signaling in human monocytes involves the phosphorylation and activation of p59hck. J. Immunol..

[bib9] Boyman O., Kovar M., Rubinstein M.P., Surh C.D., Sprent J. (2006). Selective stimulation of T cell subsets with antibody-cytokine immune complexes. Science.

[bib10] De Giovanni M., Cutillo V., Giladi A., Sala E., Maganuco C.G., Medaglia C., Di Lucia P., Bono E., Cristofani C., Consolo E. (2020). Spatiotemporal regulation of type I interferon expression determines the antiviral polarization of CD4^+^ T cells. Nat. Immunol..

[bib11] Dobin A., Davis C.A., Schlesinger F., Drenkow J., Zaleski C., Jha S., Batut P., Chaisson M., Gingeras T.R. (2013). STAR: ultrafast universal RNA-seq aligner. Bioinformatics.

[bib12] Ficht X., Iannacone M. (2020). Immune surveillance of the liver by T cells. Sci. Immunol..

[bib13] Flatz L., Hegazy A.N., Bergthaler A., Verschoor A., Claus C., Fernandez M., Gattinoni L., Johnson S., Kreppel F., Kochanek S. (2010). Development of replication-defective lymphocytic choriomeningitis virus vectors for the induction of potent CD8+ T cell immunity. Nat. Med..

[bib14] Fukao T., Koyasu S. (2000). Expression of functional IL-2 receptors on mature splenic dendritic cells. Eur. J. Immunol..

[bib15] Fumagalli V., Di Lucia P., Venzin V., Bono E.B., Jordan R., Frey C.R., Delaney W., Chisari F.V., Guidotti L.G., Iannacone M. (2020). Serum HBsAg clearance has minimal impact on CD8+ T cell responses in mouse models of HBV infection. J. Exp. Med..

[bib16] Guidotti L.G., Martinez V., Loh Y.T., Rogler C.E., Chisari F.V. (1994). Hepatitis B virus nucleocapsid particles do not cross the hepatocyte nuclear membrane in transgenic mice. J. Virol..

[bib17] Guidotti L.G., Matzke B., Schaller H., Chisari F.V. (1995). High-level hepatitis B virus replication in transgenic mice. J. Virol..

[bib18] Guidotti L.G., Rochford R., Chung J., Shapiro M., Purcell R., Chisari F.V. (1999). Viral clearance without destruction of infected cells during acute HBV infection. Science.

[bib19] Guidotti L.G., Inverso D., Sironi L., Di Lucia P., Fioravanti J., Ganzer L., Fiocchi A., Vacca M., Aiolfi R., Sammicheli S. (2015). Immunosurveillance of the liver by intravascular effector CD8(+) T cells. Cell.

[bib20] Herr F., Lemoine R., Gouilleux F., Meley D., Kazma I., Heraud A., Velge-Roussel F., Baron C., Lebranchu Y. (2014). IL-2 phosphorylates STAT5 to drive IFN-γ production and activation of human dendritic cells. J. Immunol..

[bib21] Horst A.K., Neumann K., Diehl L., Tiegs G. (2016). Modulation of liver tolerance by conventional and nonconventional antigen-presenting cells and regulatory immune cells. Cell. Mol. Immunol..

[bib53] Huang R., Grishagin I., Wang Y., Zhao T., Greene J., Obenauer J.C., Ngan D., Nguyen D.-T., Guha R., Jadhav A., Southall N., Simeonov A., Austin C.P. (2019). The NCATS BioPlanet – An Integrated Platform for Exploring the Universe of Cellular Signaling Pathways for. Toxicology, Systems Biology, and Chemical Genomics. Front Pharmacol.

[bib22] Iannacone M., Sitia G., Isogawa M., Marchese P., Castro M.G., Lowenstein P.R., Chisari F.V., Ruggeri Z.M., Guidotti L.G. (2005). Platelets mediate cytotoxic T lymphocyte-induced liver damage. Nat. Med..

[bib23] Isogawa M., Chung J., Murata Y., Kakimi K., Chisari F.V. (2013). CD40 activation rescues antiviral CD8^+^ T cells from PD-1-mediated exhaustion. PLoS Pathog..

[bib24] Jenne C.N., Kubes P. (2013). Immune surveillance by the liver. Nat. Immunol..

[bib25] Joffre O.P., Segura E., Savina A., Amigorena S. (2012). Cross-presentation by dendritic cells. Nat. Rev. Immunol..

[bib26] Kobayashi K.S., van den Elsen P.J. (2012). NLRC5: a key regulator of MHC class I-dependent immune responses. Nat. Rev. Immunol..

[bib27] Kronin V., Vremec D., Shortman K. (1998). Does the IL-2 receptor alpha chain induced on dendritic cells have a biological function?. Int. Immunol..

[bib28] Kuleshov M.V., Jones M.R., Rouillard A.D., Fernandez N.F., Duan Q., Wang Z., Koplev S., Jenkins S.L., Jagodnik K.M., Lachmann A. (2016). Enrichr: a comprehensive gene set enrichment analysis web server 2016 update. Nucleic Acids Res..

[bib29] Li B., Dewey C.N. (2011). RSEM: accurate transcript quantification from RNA-Seq data with or without a reference genome. BMC Bioinformatics.

[bib30] Li J., Lu E., Yi T., Cyster J.G. (2016). EBI2 augments Tfh cell fate by promoting interaction with IL-2-quenching dendritic cells. Nature.

[bib31] Liang D., Zuo A., Shao H., Born W.K., O’Brien R.L., Kaplan H.J., Sun D. (2012). Role of CD25+ dendritic cells in the generation of Th17 autoreactive T cells in autoimmune experimental uveitis. J. Immunol..

[bib32] Liao W., Lin J.-X., Leonard W.J. (2013). Interleukin-2 at the crossroads of effector responses, tolerance, and immunotherapy. Immunity.

[bib33] Liao Y., Smyth G.K., Shi W. (2019). The R package Rsubread is easier, faster, cheaper and better for alignment and quantification of RNA sequencing reads. Nucleic Acids Res..

[bib34] Liberzon A., Birger C., Thorvaldsdóttir H., Ghandi M., Mesirov J.P., Tamayo P. (2015). The Molecular Signatures Database (MSigDB) hallmark gene set collection. Cell Syst..

[bib35] MacParland S.A., Liu J.C., Ma X.-Z., Innes B.T., Bartczak A.M., Gage B.K., Manuel J., Khuu N., Echeverri J., Linares I. (2018). Single cell RNA sequencing of human liver reveals distinct intrahepatic macrophage populations. Nat. Commun..

[bib36] Picelli S., Faridani O.R., Björklund Å.K., Winberg G., Sagasser S., Sandberg R. (2014). Full-length RNA-seq from single cells using Smart-seq2. Nat. Protoc..

[bib37] Pol J.G., Caudana P., Paillet J., Piaggio E., Kroemer G. (2020). Effects of interleukin-2 in immunostimulation and immunosuppressionImmunomodulatory effects of IL-2. J. Exp. Med..

[bib38] Popov A., Driesen J., Abdullah Z., Wickenhauser C., Beyer M., Debey-Pascher S., Saric T., Kummer S., Takikawa O., Domann E. (2008). Infection of myeloid dendritic cells with Listeria monocytogenes leads to the suppression of T cell function by multiple inhibitory mechanisms. J. Immunol..

[bib39] Raeber M.E., Rosalia R.A., Schmid D., Karakus U., Boyman O. (2020). Interleukin-2 signals converge in a lymphoid-dendritic cell pathway that promotes anticancer immunity. Sci. Transl. Med..

[bib40] Ramachandran P., Dobie R., Wilson-Kanamori J.R., Dora E.F., Henderson B.E.P., Luu N.T., Portman J.R., Matchett K.P., Brice M., Marwick J.A. (2019). Resolving the fibrotic niche of human liver cirrhosis at single-cell level. Nature.

[bib41] Ritchie M.E., Phipson B., Wu D., Hu Y., Law C.W., Shi W., Smyth G.K. (2015). limma powers differential expression analyses for RNA-sequencing and microarray studies. Nucleic Acids Res..

[bib42] Robinson M.D., McCarthy D.J., Smyth G.K. (2010). edgeR: a Bioconductor package for differential expression analysis of digital gene expression data. Bioinformatics.

[bib43] Sitia G., Iannacone M., Aiolfi R., Isogawa M., van Rooijen N., Scozzesi C., Bianchi M.E., von Andrian U.H., Chisari F.V., Guidotti L.G. (2011). Kupffer cells hasten resolution of liver immunopathology in mouse models of viral hepatitis. PLoS Pathog..

[bib44] Stuart T., Butler A., Hoffman P., Hafemeister C., Papalexi E., Mauck W.M., Hao Y., Stoeckius M., Smibert P., Satija R. (2019). Comprehensive Integration of Single-Cell Data. Cell.

[bib45] Subramanian A., Tamayo P., Mootha V.K., Mukherjee S., Ebert B.L., Gillette M.A., Paulovich A., Pomeroy S.L., Golub T.R., Lander E.S., Mesirov J.P. (2005). Gene set enrichment analysis: a knowledge-based approach for interpreting genome-wide expression profiles. Proc. Natl. Acad. Sci. U S A.

[bib46] Vollmar B., Menger M.D. (2009). The hepatic microcirculation: mechanistic contributions and therapeutic targets in liver injury and repair. Physiol. Rev..

[bib47] Warren A., Le Couteur D.G., Fraser R., Bowen D.G., McCaughan G.W., Bertolino P. (2006). T lymphocytes interact with hepatocytes through fenestrations in murine liver sinusoidal endothelial cells. Hepatology.

[bib48] West E.E., Jin H.-T., Rasheed A.-U., Penaloza-Macmaster P., Ha S.-J., Tan W.G., Youngblood B., Freeman G.J., Smith K.A., Ahmed R. (2013). PD-L1 blockade synergizes with IL-2 therapy in reinvigorating exhausted T cells. J. Clin. Invest..

[bib49] Wong Y.C., Tay S.S., McCaughan G.W., Bowen D.G., Bertolino P. (2015). Immune outcomes in the liver: Is CD8 T cell fate determined by the environment?. J. Hepatol..

[bib50] Wu X., Hollingshead N., Roberto J., Knupp A., Kenerson H., Chen A., Strickland I., Horton H., Yeung R., Soysa R., Crispe I.N. (2020). Human liver macrophage subsets defined by CD32. Front. Immunol..

[bib51] Wuest S.C., Edwan J.H., Martin J.F., Han S., Perry J.S.A., Cartagena C.M., Matsuura E., Maric D., Waldmann T.A., Bielekova B. (2011). A role for interleukin-2 trans-presentation in dendritic cell-mediated T cell activation in humans, as revealed by daclizumab therapy. Nat. Med..

[bib52] Zordan P., Cominelli M., Cascino F., Tratta E., Poliani P.L., Galli R. (2018). Tuberous sclerosis complex-associated CNS abnormalities depend on hyperactivation of mTORC1 and Akt. J. Clin. Invest..

