# Supplementary material for: Identification of a Kupffer cell subset capable of reverting the T cell dysfunction induced by hepatocellular priming
Source: Immunity. 2021 Sep 14;54(9):2089–2100.e8. doi: 10.1016/j.immuni.2021.05.005 (PMC8459394; doi:10.1016/j.immuni.2021.05.005)
Supplement: Document S1. Figures S1–S5 [file mmc1.pdf]

Immunity, Volume *54*

Supplemental information

Identification of a Kupffer cell subset capable of reverting the T cell dysfunction induced by hepatocellular priming

Giorgia De Simone, Francesco Andreata, Camille Bleriot, Valeria Fumagalli, Chiara Laura, José M. Garcia-Manteiga, Pietro Di Lucia, Stefano Gilotto, Xenia Ficht, Federico F. De Ponti, Elisa B. Bono, Leonardo Giustini, Gioia Ambrosi, Marta Mainetti, Paola Zordan, Alexandre P. Bénéchet, Micol Ravà, Svetoslav Chakarov, Federica Moalli, Marc Bajenoff, Luca G. Guidotti, Florent Ginhoux, and Matteo Iannacone

Figure S1

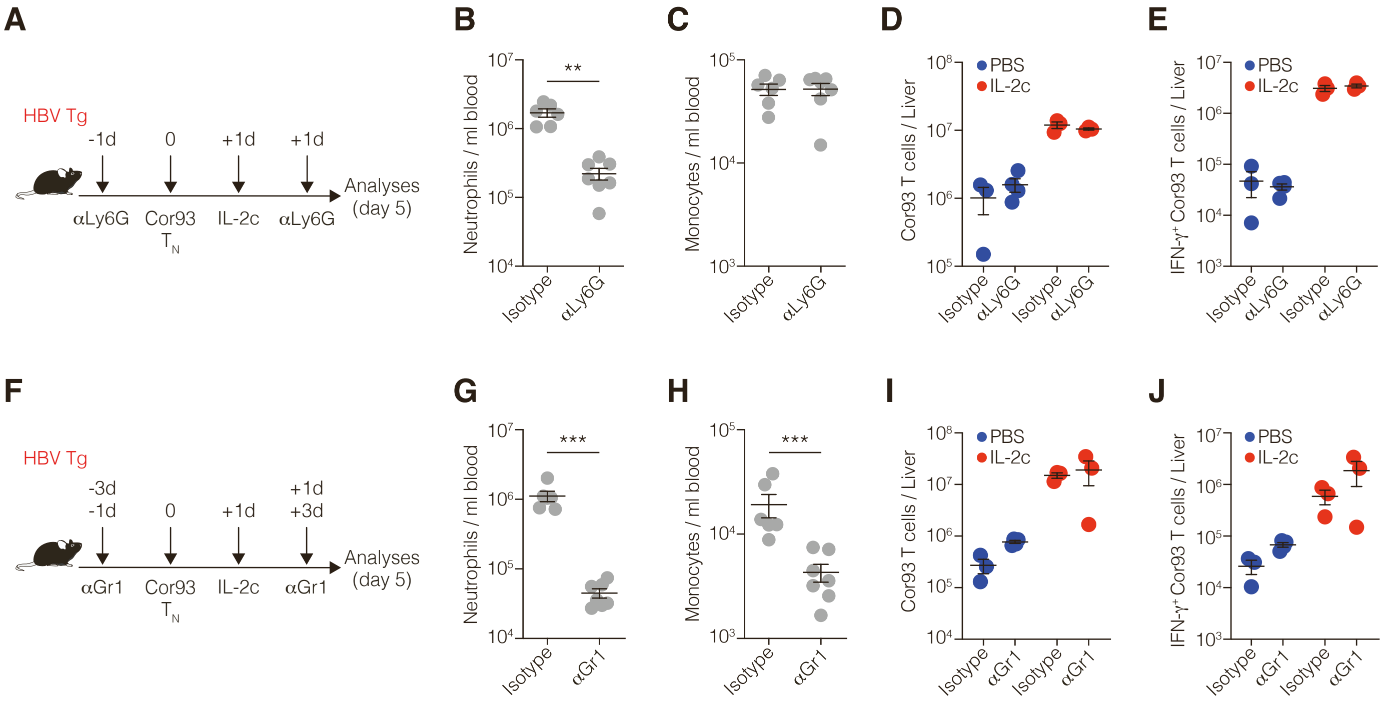

Figure S1. Neutrophils and monocytes are dispensable for T cell reinvigoration by IL-2. Related to Figure 1

(**A**) Schematic representation of the experimental setup. 1 x 10^6 Cor93 T_N were transferred into HBV transgenic (HBV Tg) recipients. Mice were injected with anti-Ly6G depleting antibody or the isotype control one day before and one day after T cell injection. Indicated mice received IL-2c one day after Cor93 T_N transfer. Livers were collected and analyzed five days after T cell transfer.
(**B-C**) Numbers of neutrophils (**B**) and monocytes (**C**) in the blood in the indicated mice at the time of Cor93 T_N injection (Isotype control n=6, anti-Ly6G n=8). **p value<0.01, one tailed Mann-Whitney U-test.
(**D-E**) Total numbers (**D**) and numbers of IFN-γ-producing (**E**) Cor93 T cells in the livers of the indicated mice (PBS: isotype control n=3, anti-Ly6G n=4; IL-2c: isotype control n=3, anti-Ly6G n=3).
(**F**) Schematic representation of the experimental setup. 1 x 10^6 Cor93 T_N were transferred into HBV Tg recipients. Mice were injected with anti-Gr1 depleting antibody or isotype control every 48h starting 3 days before T cell injection. Indicated mice received IL-2c one day after Cor93 T_N cell transfer. Livers were collected and analyzed five days after T cell transfer.
(**G-H**) Numbers of neutrophils (**G**) and monocytes (**H**) in the blood of the indicated mice at the time of T cell injection (Isotype control n=6, anti-Gr1 n=7). *** p value<0.001, one-tailed Mann-Whitney U-test.
(**I-J**) Total numbers (**I**) and numbers of IFN-γ-producing (**J**) Cor93 T cells in the livers of the indicated mice (PBS: isotype control n=3, anti-Gr1 n=4; IL-2c: isotype control n=3 anti-Gr1, n=3). Data are representative of at least 3 independent experiments.

Figure S2

A

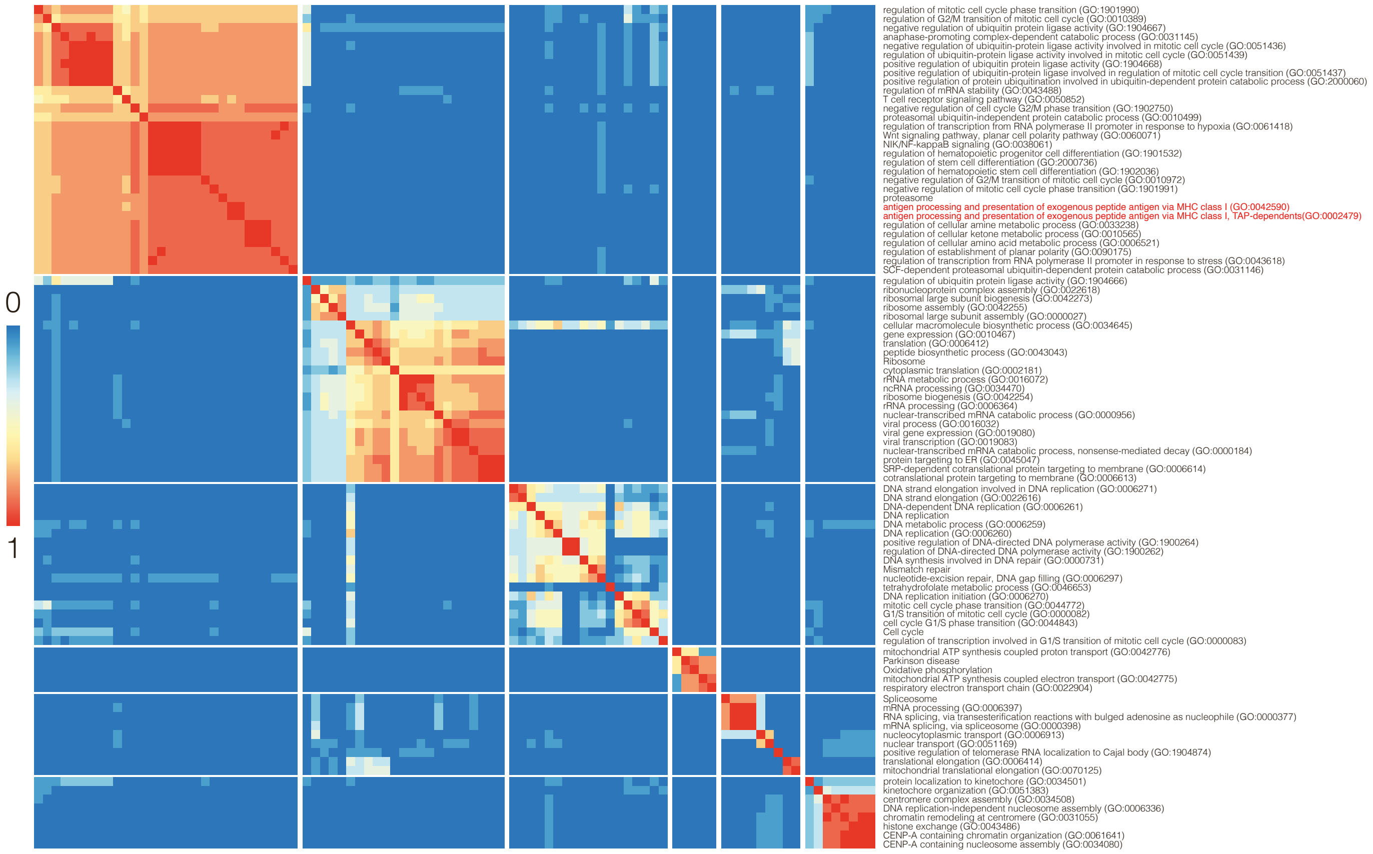
0
1
regulation of mitotic cell cycle phase transition (GO:1901990)
regulation of G2/M transition of mitotic cell cycle (GO:0010389)
negative regulation of ubiquitin protein ligase activity (GO:1904667)
anaphase-promoting complex-dependent catabolic process (GO:0031145)
negative regulation of ubiquitin-protein ligase activity involved in mitotic cell cycle (GO:0051436)
regulation of ubiquitin-protein ligase activity involved in mitotic cell cycle (GO:0051439)
positive regulation of ubiquitin protein ligase activity (GO:1904668)
positive regulation of ubiquitin-protein ligase involved in regulation of mitotic cell cycle transition (GO:0051437)
positive regulation of protein ubiquitination involved in ubiquitin-dependent protein catabolic process (GO:2000060)
regulation of mRNA stability (GO:0043488)
T cell receptor signaling pathway (GO:0050852)
negative regulation of cell cycle G2/M phase transition (GO:1902750)
proteasomal ubiquitin-independent protein catabolic process (GO:0010499)
regulation of transcription from RNA polymerase II promoter in response to hypoxia (GO:0061418)
Wnt signaling pathway, planar cell polarity pathway (GO:0060071)
NIK/NF-kappaB signaling (GO:0038061)
regulation of hematopoietic progenitor cell differentiation (GO:1901532)
regulation of stem cell differentiation (GO:2000736)
regulation of hematopoietic stem cell differentiation (GO:1902036)
negative regulation of G2/M transition of mitotic cell cycle (GO:0010972)
negative regulation of mitotic cell cycle phase transition (GO:1901991)
proteasome
antigen processing and presentation of exogenous peptide antigen via MHC class I (GO:0042590)
antigen processing and presentation of exogenous peptide antigen via MHC class I, TAP-dependents(GO:0002479)
regulation of cellular amine metabolic process (GO:0033238)
regulation of cellular ketone metabolic process (GO:0010565)
regulation of cellular amino acid metabolic process (GO:0006521)
regulation of establishment of planar polarity (GO:0090175)
regulation of transcription from RNA polymerase II promoter in response to stress (GO:0043618)
SCF-dependent proteasomal ubiquitin-dependent protein catabolic process (GO:0031146)
regulation of ubiquitin protein ligase activity (GO:1904666)
ribonucleoprotein complex assembly (GO:0022618)
ribosomal large subunit biogenesis (GO:0042273)
ribosome assembly (GO:0042255)
ribosomal large subunit assembly (GO:0000027)
cellular macromolecule biosynthetic process (GO:0034645)
gene expression (GO:0010467)
translation (GO:0006412)
peptide biosynthetic process (GO:0043043)
Ribosome
cytoplasmic translation (GO:0002181)
rRNA metabolic process (GO:0016072)
ncRNA processing (GO:0034470)
ribosome biogenesis (GO:0042254)
rRNA processing (GO:0006364)
nuclear-transcribed mRNA catabolic process (GO:0000956)
viral process (GO:0016032)
viral gene expression (GO:0019080)
viral transcription (GO:0019083)
nuclear-transcribed mRNA catabolic process, nonsense-mediated decay (GO:0000184)
protein targeting to ER (GO:0045047)
SRP-dependent cotranslational protein targeting to membrane (GO:0006614)
cotranslational protein targeting to membrane (GO:0006613)
DNA strand elongation involved in DNA replication (GO:0006271)
DNA strand elongation (GO:0022616)
DNA-dependent DNA replication (GO:0006261)
DNA replication
DNA metabolic process (GO:0006259)
DNA replication (GO:0006260)
positive regulation of DNA-directed DNA polymerase activity (GO:1900264)
regulation of DNA-directed DNA polymerase activity (GO:1900262)
DNA synthesis involved in DNA repair (GO:0000731)
Mismatch repair
nucleotide-excision repair, DNA gap filling (GO:0006297)
tetrahydrofolate metabolic process (GO:0046653)
DNA replication initiation (GO:0006270)
mitotic cell cycle phase transition (GO:0044772)
G1/S transition of mitotic cell cycle (GO:0000082)
cell cycle G1/S phase transition (GO:0044843)
Cell cycle
regulation of transcription involved in G1/S transition of mitotic cell cycle (GO:0000083)
mitochondrial ATP synthesis coupled proton transport (GO:0042776)
Parkinson disease
Oxidative phosphorylation
mitochondrial ATP synthesis coupled electron transport (GO:0042775)
respiratory electron transport chain (GO:0022904)
Spliceosome
mRNA processing (GO:0006397)
RNA splicing, via transesterification reactions with bulged adenosine as nucleophile (GO:0000377)
mRNA splicing, via spliceosome (GO:0000398)
nucleocytoplasmic transport (GO:0006913)
nuclear transport (GO:0051169)
positive regulation of telomerase RNA localization to Cajal body (GO:1904874)
translational elongation (GO:0006414)
mitochondrial translational elongation (GO:0070125)
protein localization to kinetochore (GO:0034501)
kinetochore organization (GO:0051383)
centromere complex assembly (GO:0034508)
DNA replication-independent nucleosome assembly (GO:0006336)
chromatin remodeling at centromere (GO:0031055)
histone exchange (GO:0043486)
CENP-A containing chromatin organization (GO:0061641)
CENP-A containing nucleosome assembly (GO:0034080)

B

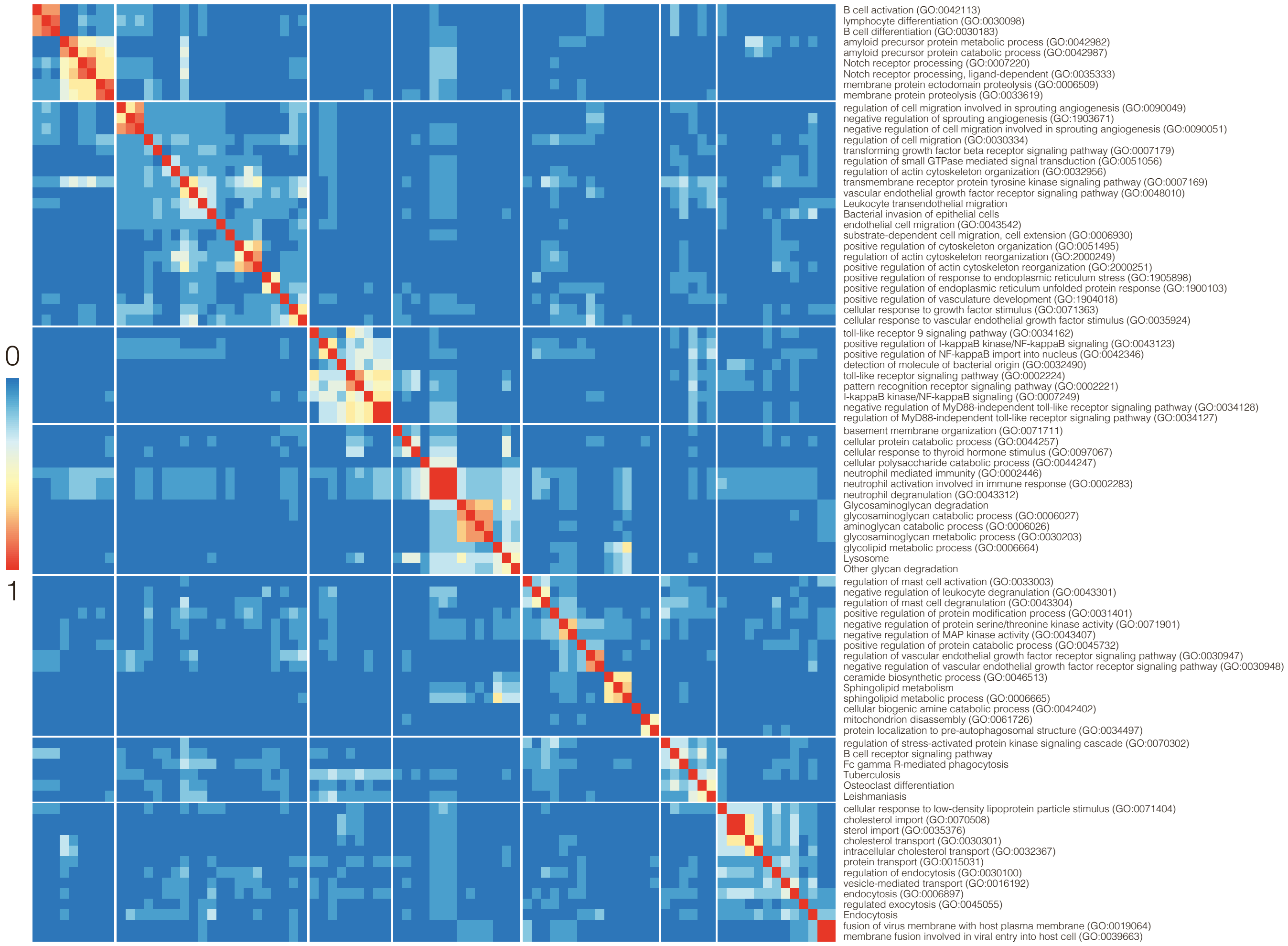
0
1
B cell activation (GO:0042113)
lymphocyte differentiation (GO:0030098)
B cell differentiation (GO:0030183)
amyloid precursor protein metabolic process (GO:0042982)
amyloid precursor protein catabolic process (GO:0042987)
Notch receptor processing (GO:0007220)
Notch receptor processing, ligand-dependent (GO:0035333)
membrane protein ectodomain proteolysis (GO:0006509)
membrane protein proteolysis (GO:0033619)
regulation of cell migration involved in sprouting angiogenesis (GO:0090049)
negative regulation of sprouting angiogenesis (GO:1903671)
negative regulation of cell migration involved in sprouting angiogenesis (GO:0090051)
regulation of cell migration (GO:0030334)
transforming growth factor beta receptor signaling pathway (GO:0007179)
regulation of small GTPase mediated signal transduction (GO:0051056)
regulation of actin cytoskeleton organization (GO:0032956)
transmembrane receptor protein tyrosine kinase signaling pathway (GO:0007169)
vascular endothelial growth factor receptor signaling pathway (GO:0048010)
Leukocyte transendothelial migration
Bacterial invasion of epithelial cells
endothelial cell migration (GO:0043542)
substrate-dependent cell migration, cell extension (GO:0006930)
positive regulation of cytoskeleton organization (GO:0051495)
regulation of actin cytoskeleton reorganization (GO:2000249)
positive regulation of actin cytoskeleton reorganization (GO:2000251)
positive regulation of response to endoplasmic reticulum stress (GO:1905898)
positive regulation of endoplasmic reticulum unfolded protein response (GO:1900103)
positive regulation of vasculature development (GO:1904018)
cellular response to growth factor stimulus (GO:0071363)
cellular response to vascular endothelial growth factor stimulus (GO:0035924)
toll-like receptor 9 signaling pathway (GO:0034162)
positive regulation of I-kappaB kinase/NF-kappaB signaling (GO:0043123)
positive regulation of NF-kappaB import into nucleus (GO:0042346)
detection of molecule of bacterial origin (GO:0032490)
toll-like receptor signaling pathway (GO:0002224)
pattern recognition receptor signaling pathway (GO:0002221)
I-kappaB kinase/NF-kappaB signaling (GO:0007249)
negative regulation of MyD88-independent toll-like receptor signaling pathway (GO:0034128)
regulation of MyD88-independent toll-like receptor signaling pathway (GO:0034127)
basement membrane organization (GO:0071711)
cellular protein catabolic process (GO:0044257)
cellular response to thyroid hormone stimulus (GO:0097067)
cellular polysaccharide catabolic process (GO:0044247)
neutrophil mediated immunity (GO:0002446)
neutrophil activation involved in immune response (GO:0002283)
neutrophil degranulation (GO:0043312)
Glycosaminoglycan degradation
glycosaminoglycan catabolic process (GO:0006027)
aminoglycan catabolic process (GO:0006026)
glycosaminoglycan metabolic process (GO:0030203)
glycolipid metabolic process (GO:0006664)
Lysosome
Other glycan degradation
regulation of mast cell activation (GO:0033003)
negative regulation of leukocyte degranulation (GO:0043301)
regulation of mast cell degranulation (GO:0043304)
positive regulation of protein modification process (GO:0031401)
negative regulation of protein serine/threonine kinase activity (GO:0071901)
negative regulation of MAP kinase activity (GO:0043407)
positive regulation of protein catabolic process (GO:0045732)
regulation of vascular endothelial growth factor receptor signaling pathway (GO:0030947)
negative regulation of vascular endothelial growth factor receptor signaling pathway (GO:0030948)
ceramide biosynthetic process (GO:0046513)
Sphingolipid metabolism
sphingolipid metabolic process (GO:0006665)
cellular biogenic amine catabolic process (GO:0042402)
mitochondrion disassembly (GO:0061726)
protein localization to pre-autophagosomal structure (GO:0034497)
regulation of stress-activated protein kinase signaling cascade (GO:0070302)
B cell receptor signaling pathway
Fc gamma R-mediated phagocytosis
Tuberculosis
Osteoclast differentiation
Leishmaniasis
cellular response to low-density lipoprotein particle stimulus (GO:0071404)
cholesterol import (GO:0070508)
sterol import (GO:0035376)
cholesterol transport (GO:0030301)
intracellular cholesterol transport (GO:0032367)
protein transport (GO:0015031)
regulation of endocytosis (GO:0030100)
vesicle-mediated transport (GO:0016192)
endocytosis (GO:0006897)
regulated exocytosis (GO:0045055)
Endocytosis
fusion of virus membrane with host plasma membrane (GO:0019064)
membrane fusion involved in viral entry into host cell (GO:0039663)

Figure S2. Regulated processes in KCs upon in vivo IL-2c treatment. Related to Figure 2

(**A**) Clustering of top significant (EnrichR Combined Score > 100, FDR < 0.05) Gene Ontology Biological Processes and Kyoto Encyclopedia of Genes and Genomes (KEGG) pathways of biological processes up-regulated in KCs upon in vivo IL-2c treatment. The thermal scale represents the Jaccard Similarity Coefficient between every gene set pair (blue representing a 0 Similarity Coefficient, red a 1 Similarity Coefficient).

(**B**) Clustering of top significant (EnrichR Combined Score > 30, FDR<0.05) Gene Ontology Biological Processes and KEGG pathways of biological processes down-regulated in KCs upon in vivo IL-2c treatment. The thermal scale represents the Jaccard Similarity Coefficient between every gene set pair (blue representing a 0 Similarity Coefficient, red a 1 Similarity Coefficient).

Figure S3

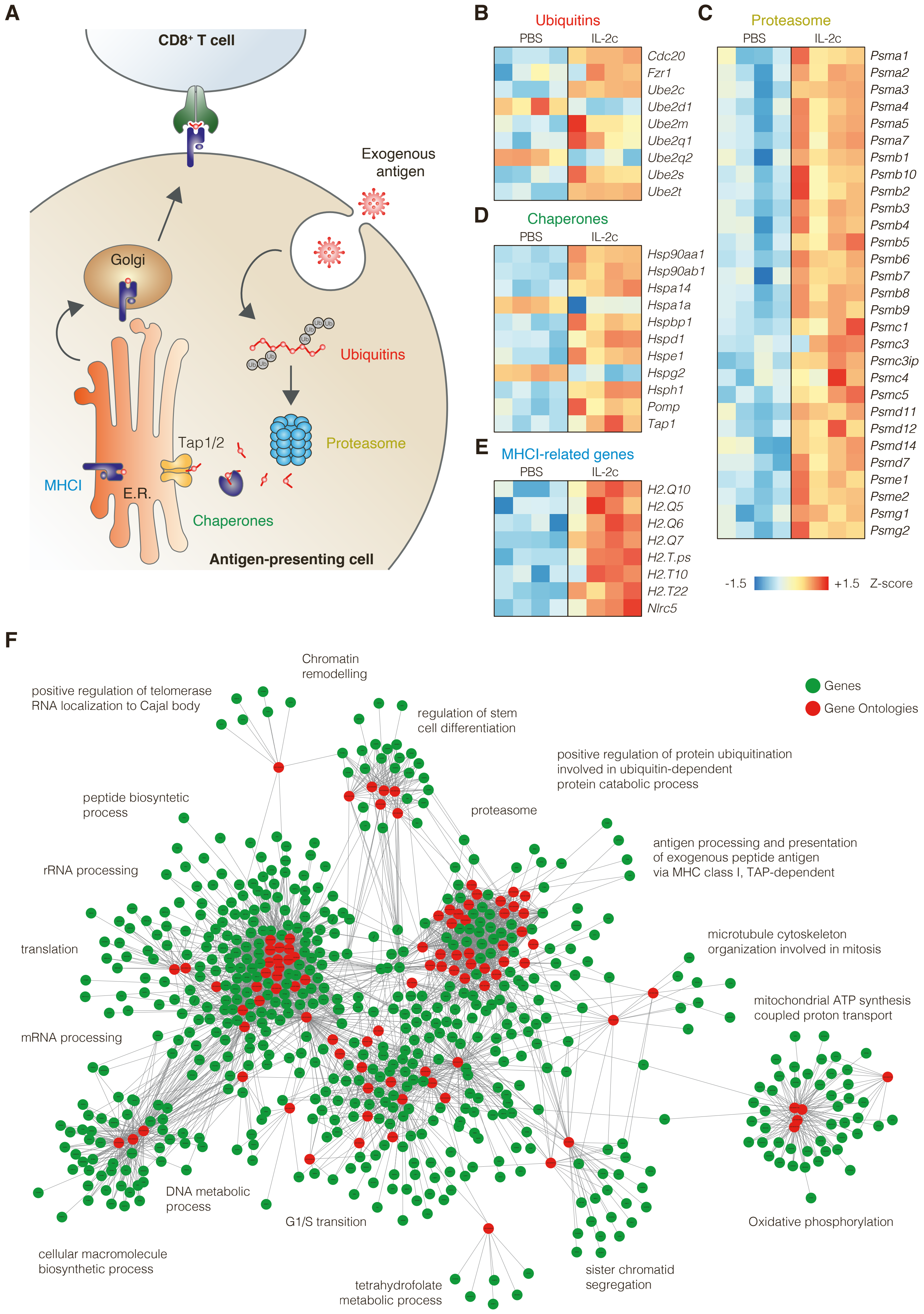
A
CD8+ T cell
Exogenous antigen
Golgi
Ubiquitins
Tap1/2
Proteasome
MHCI
E.R.
Chaperones
Antigen-presenting cell
B
Ubiquitins
PBS
IL-2c
Cdc20
Fzr1
Ube2c
Ube2d1
Ube2m
Ube2q1
Ube2q2
Ube2s
Ube2t
C
Proteasome
PBS
IL-2c
Psma1
Psma2
Psma3
Psma4
Psma5
Psma7
Psmb1
Psmb10
Psmb2
Psmb3
Psmb4
Psmb5
Psmb6
Psmb7
Psmb8
Psmb9
Psmc1
Psmc3
Psmc3ip
Psmc4
Psmc5
Psmd11
Psmd12
Psmd14
Psmd7
Psme1
Psme2
Psmg1
Psmg2
D
Chaperones
PBS
IL-2c
Hsp90aa1
Hsp90ab1
Hspa14
Hspa1a
Hspbp1
Hspd1
Hspe1
Hspg2
Hsph1
Pomp
Tap1
E
MHCI-related genes
PBS
IL-2c
H2.Q10
H2.Q5
H2.Q6
H2.Q7
H2.T.ps
H2.T10
H2.T22
Nlrc5
-1.5
+1.5
Z-score
F
Chromatin remodelling
positive regulation of telomerase RNA localization to Cajal body
regulation of stem cell differentiation
Genes
Gene Ontologies
positive regulation of protein ubiquitination involved in ubiquitin-dependent protein catabolic process
peptide biosyntetic process
proteasome
antigen processing and presentation of exogenous peptide antigen via MHC class I, TAP-dependent
rRNA processing
translation
microtubule cytoskeleton organization involved in mitosis
mitochondrial ATP synthesis coupled proton transport
mRNA processing
DNA metabolic process
G1/S transition
Oxidative phosphorylation
cellular macromolecule biosynthetic process
tetrahydrofolate metabolic process
sister chromatid segregation

Figure S3. Genes associated to cross-presentation are upregulated in KCs upon *in vivo* IL-2c treatment. Related to Figure 2

(**A-E**) Schematic representation (**A**) and expression heatmap (**B-E**) of selected genes belonging to biological processes implicated in antigen cross-presentation, upregulated in KCs after IL-2c treatment. Values are in Z-score, calculated from scaling by row the Log2(TPM) values.
(**F**) Cytoscape network of top significant (EnrichR Combined Score > 100, FDR < 0.05) Gene Ontology Biological Processes and KEGG pathways of up-regulated processes. Red dots indicate enriched terms, green dots indicate the relative enriched genes.

Figure S4

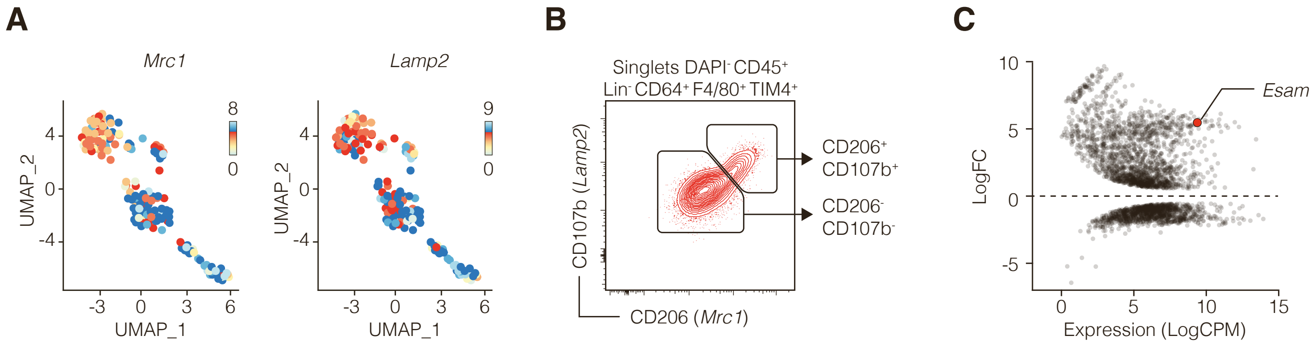

Figure S4. ***Ad interim*** **bulk RNA sequencing on sorted KC subpopulations for the identification of suitable markers. Related to Figure 3**

(**A**) Feature plot representation of the normalized expression level of *Mrc1* (CD206) and *Lamp2* (CD107b) on the scRNAseq dataset described in Fig. 3. The expression is measured as the ln (TPM+1).
(**B**) Sorting strategy for ad interim bulk RNA sequencing.
(**C**) MA plot showing differentially expressed genes (DEGs, p value < 0.001) between CD206$^-$ CD107b$^-$ KCs and CD206$^+$ CD107b$^+$ KCs. Each dot denotes a differentially expressed gene. The y-axis represents the logarithmic Fold Change (FC) between CD206$^-$ CD107b$^-$ KCs and CD206$^+$ CD107b$^+$ KCs, while the x-axis shows the expression level of each DEG in logarithmic Counts Per Million (CPM). *Esam* is highlighted as one of the most over-expressed gene in CD206$^+$ CD107b$^+$ KCs compared to CD206$^-$ CD107b$^-$ KCs.

Figure S5

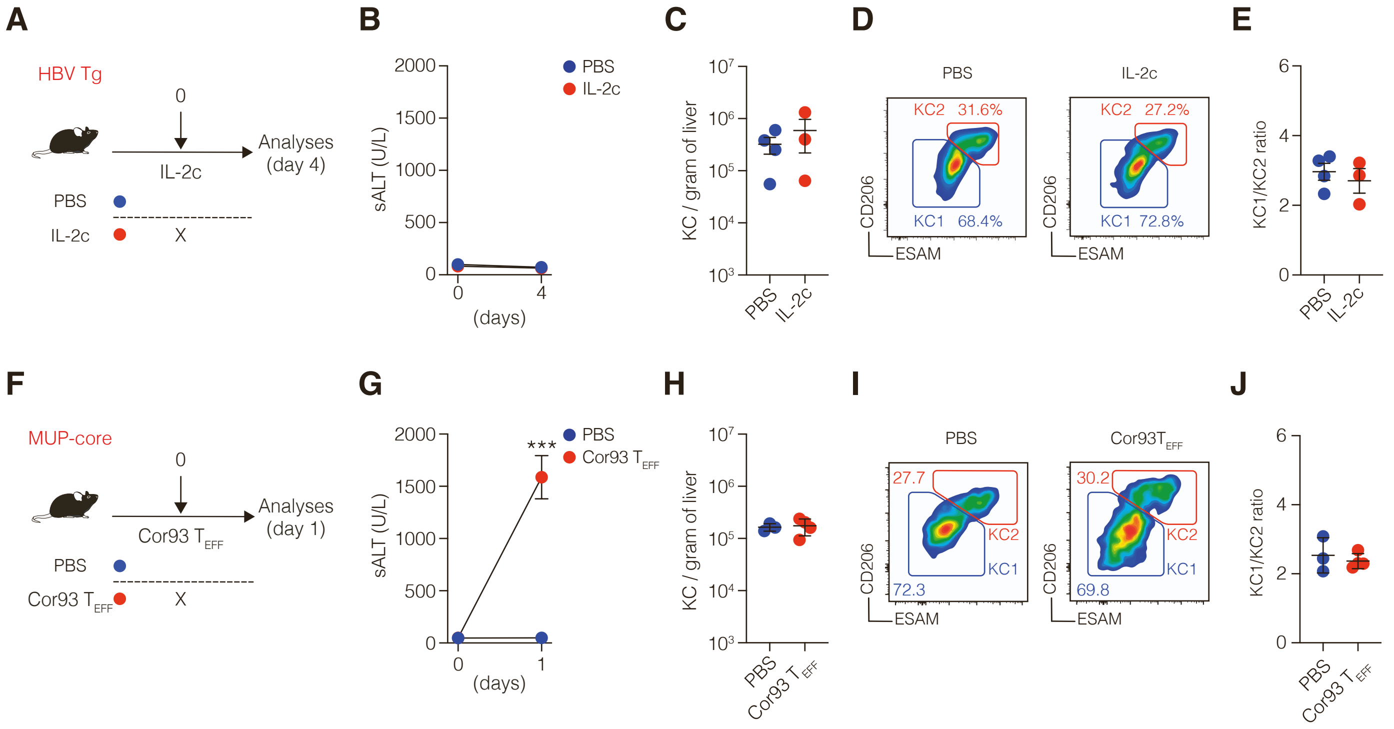

Figure S5. IL-2c treatment alone or liver inflammation have no impact on KC1/KC2 ratio. Related to Figure 4

(**A**) Schematic representation of the experimental setup. HBV Tg mice were treated with PBS or IL-2c and livers were collected and analyzed four days after treatment.
(**B**) Amount of ALT in the serum of the indicated mice at the indicated timepoints.
(**C**) Numbers of KCs (identified as live, $CD45^+$, $F4/80^+$, $TIM4^+$ cells) per gram of liver in the indicated mice.
(**D**) Representative flow cytometry plots of KC1 ($CD206^-$ $ESAM^-$) and KC2 ($CD206^+$ $ESAM^+$) in the indicated mice.
(**E**) KC1/KC2 ratio in the indicated mice (PBS, n=4; IL-2c, n=3).
(**F**) Schematic representation of the experimental setup. MUP-core mice were injected with PBS or Cor93 T_{EFF}. Livers were collected and analyzed one day after T cell transfer.
(**G**) Amount of ALT in the serum of indicated mice at the indicated timepoints.
(**H**) Numbers of KCs per gram of liver in the indicated mice.
*** p value< 0.001, two-way ANOVA with Sidak's multiple comparison test.
(**I**) Representative flow cytometry plots of KC1 ($CD206^-$ $ESAM^-$) and KC2 ($CD206^+$ $ESAM^+$) in the indicated mice.
(**J**) KC1/KC2 ratio in the indicated mice (PBS, n=3; Cor93 T_{eff}, n=3). Data are representative of at least 2 independent experiments.
